# Mechanisms of Shock Dissipation in Semicrystalline Polyethylene

**DOI:** 10.3390/polym15214262

**Published:** 2023-10-30

**Authors:** John P. Mikhail, Gregory C. Rutledge

**Affiliations:** 1 Department of Chemical Engineering, Massachusetts Institute of Technology, 77 Massachusetts Avenue, Cambridge, MA 02139, USA; 2 Institute for Soldier Nanotechnologies, Massachusetts Institute of Technology, 500 Technology Square, Cambridge, MA 02139, USA

**Keywords:** molecular simulation, semicrystalline, polyethylene, shock, deformation mechanism, slip, kink band

## Abstract

Semicrystalline polymers are lightweight, multiphase materials that exhibit attractive shock dissipation characteristics and have potential applications as protective armor for people and equipment. For shocks of 10 GPa or less, we analyzed various mechanisms for the storage and dissipation of shock wave energy in a realistic, united atom (UA) model of semicrystalline polyethylene. Systems characterized by different levels of crystallinity were simulated using equilibrium molecular dynamics with a Hugoniostat to ensure that the resulting states conform to the Rankine–Hugoniot conditions. To determine the role of structural rearrangements, order parameters and configuration time series were collected during the course of the shock simulations. We conclude that the major mechanisms responsible for the storage and dissipation of shock energy in semicrystalline polyethylene are those associated with plastic deformation and melting of the crystalline domain. For this UA model, plastic deformation occurs primarily through fine crystallographic slip and the formation of kink bands, whose long period decreases with increasing shock pressure.

## 1. Introduction

Shock waves are supersonic, high pressure waves that propagate through a material as a result of an extreme deformation or disturbance [1,2]. They are encountered in military settings, resulting from ballistic or explosive impact, and pose major safety hazards to people and equipment. Additionally, they are an important safety consideration when designing supersonic aircraft [3] and in controlling ignition or pressure waves from certain chemical processes [4,5]. The design of materials that are capable of withstanding and dissipating the energy from these shock waves decreases the danger to the user; however, many traditional materials are incapable of either dissipating the shock energy effectively or maintaining their structural integrity after shock for continued use. For this purpose, polymeric materials offer a promising area of design due to their wide diversity of useful material properties, a result of flexibility in both chemical composition and molecular organization.

The design of materials capable of withstanding extreme shock pressure requires knowledge of the relevant shock dissipation mechanisms, in order to anticipate the amount of energy that can be absorbed by the material. At high shock pressures, chemical dissociation is a significant mechanism for energy dissipation. In fact, there are certain chemical reaction pathways that are unique to shock events [6]. At low shock pressures below the threshold for breaking chemical bonds, other essentially thermophysical mechanisms to dissipate energy must be activated. For example, simulations of shocked diblock copolymers in a lamellar morphology revealed that the polymers can absorb the energy of a shock wave by decreasing the segregation of their initially distinct phases [7].

Shock waves induce nonlinear responses in materials due to the extreme pressure and temperature applied, complicating a mechanistic analysis. The first step in understanding shock response is the construction of an equation of state for a particular material; this equation gives the relationship between pressure and a specific volume, or between the shock velocity and the particle velocity, for a material undergoing shock deformation. The velocities can be derived from the pressure–volume description using the Rankine–Hugoniot (RH) conditions, which describe the relationship between states on either side of the shock wave [1,2,8]. The RH conditions are [8]

(1) $$\rho_{0}u_{s}=\rho \left(u_{s}-u_{p}\right)$$

(2) $$P_{zz}-P_{0}=\rho_{0}u_{s}u_{p}$$

(3) $$\Delta E=\frac{{(P}_{zz}+P_{0})(v_{0}-v)}{2},$$

where the subscript 0 designates the unshocked, or pre-shocked, state. *ρ* is the density, *v* = *ρ*^−1^ is the specific volume, *P_zz_* = *−*σ_zz_ where σ is the stress tensor and the subscript *zz* indicates the normal component of the stress tensor in the direction of the shock wave, in this case in the *z*-plane. *u_p_* is the particle velocity, *u_s_* is the shock velocity, and Δ*E* is the change in total internal energy as a result of the shock. For typical shock pressures, *P_zz_* − *P_0_* is well approximated as simply *P_zz_*.

Pressure–volume relationships associated with shock in a specific material can be measured experimentally, and they can be estimated theoretically or computationally. Nonequilibrium molecular dynamics (NEMD) is typically used to simulate systems under the application of a driving force such as a piston colliding with the system and forming a shock wave from the resulting impact. Equilibrium simulation methods have also been developed to study state points along the Hugoniot, a curve that satisfies the RH conditions for all points along the curve. Examples include the *NP_zz_*Hug method of Ravelo et al. [8] and the Multiscale Shock Technique (MSST) of Reed et al. [9], each of which modify the equations of motion to simulate a shocked system at equilibrium that lies on the Hugoniot. The *NP_zz_*Hug method of Ravelo, used in this work, employs a “uniaxial Hugoniostat,” similar to a single (non-chain) Nosé–Hoover thermostat and barostat [10] in which the target pressure is specified and the target energy at each time step is computed as a function of the current configuration; the equilibrium value of the target energy is also consistent with the RH conditions [8].

Semicrystalline polyethylene (e.g., Dyneema^®^ or Spectra^®^) is a material that is commonly used in soft and hard body armor because it can be spun into fibers with exceptionally high specific strength and specific modulus, resulting in lightweight fabrics that can be cut and sewn or laminated as reinforcing elements in composites. Polyethylene is widely used in engineering materials to withstand extreme impacts; gel-spun polyethylene strands have stiffnesses comparable to that of steel, while maintaining light weight and ease of manufacturing [11]. Other applications of polyethylene that take advantage of its high strength and toughness include the structural engineering of aerospace and military components [12], packaging of consumer products, films, water and gas pipelines [13], and components of artificial joints [14]. Polyethylene is also the prototype for many other semicrystalline polymers. On the length scale of micrometers, semicrystalline polymers comprise domains of both crystalline and noncrystalline materials, which differ in their mechanical compliances (ease of deformation under an applied stress) and can contribute to the dissipation of energy during shock wave progression. At the nanoscale, the representative motif of the system consists of alternating layers of crystalline and noncrystalline material. Mechanical properties vary with the thickness of the crystalline lamellae [15,16]. Importantly, covalently bonded chains weave back and forth between crystalline and noncrystalline domains, giving rise to a unique interfacial region called the “interphase,” in which the constraints of connection to the crystalline domain strongly influence the topology of the chains [17], making this region distinct from an amorphous melt or glass. Chains in the noncrystalline domain consist of loops (chain segments with typically non-adjacent connections to the same crystal lamella), tails (chain segments that connect to the lamella at one end and terminate in the noncrystalline domain at the other end), and bridges (chain segments that traverse the noncrystalline domain to connect to distinct lamellae). For the purposes of modeling the coupling between crystalline and noncrystalline domains, the simplest representative volume element that includes both types of domains is the “lamellar stack” model [18].

The first simulations of semicrystalline polyethylene were those reported by in’t Veld et al. [19] using the Interphase Monte Carlo (IMC) method [16,17] to sample the distributions of loops, tails, and bridges in a thermodynamically consistent manner. In that method, nonlocal reptation and end-bridging moves were introduced to sample different topologies within a single Monte Carlo simulation. The resulting configurations were then used in a series of studies of isothermal deformations [20,21,22,23,24,25]. For nonisothermal deformations like shock, chemical as well as thermophysical rearrangements can occur at sufficiently high pressure, necessitating the use of bond-breaking methods like density functional theory (DFT) or reactive force fields such as ReaxFF [26] or AIREBO-M [27]. Shock studies of PE using DFT simulations have been used to obtain chemical [28] and thermodynamic [29] information for shock pressures up to 250 GPa. Typically, simulations of shock waves in polyethylene consider crystalline and noncrystalline domains separately. Elder et al. [30] first considered semicrystalline polyethylene (SCPE) models that comprised the two types of domains together, using a method involving *deletion*, *cutting,* and *melting* (DCM) to reduce density and introduce conformational disorder to the noncrystalline domain. They then used NEMD simulations to investigate how the interfaces between crystalline and noncrystalline domains of SCPE transmit and reflect propagating shock waves, based on the impedance of each region. The DCM method is analogous in many respects to the IMC method, except that it lacks the ability to sample alternative connectivities efficiently once the initial structure is generated. As a result, the interphase topology obtained does not minimize free energy. The DCM method also retains some memory in the noncrystalline region of the crystalline region from which it was generated. It remains an open question whether the shock response of a semicrystalline polymer is sensitive to the topological nature of the interphase.

Crystalline regions in general deform through a variety of mechanisms, including defect-mediated mechanisms (slip, kinking, twinning, Martensitic transformation, etc.) and melting–recrystallization [21,31]. Deformation of noncrystalline regions are relatively simpler, only straining due to interlamellar compression and shear. Previous studies of crystalline polyethylene have found that the (100)[001] and (100)[010] fine crystallographic slip mechanisms are dominant in compression because they have the lowest activation energy barriers [31]. The notation (*hkl*)[*uvw*] refers to slip in the (*hkl*) plane and [*uvw*] direction, where *h*, *k*, *l*, *u*, *v*, and *w* are Miller indices. Galeski et al. showed that, for plane strain compression of high-density polyethylene (HDPE), spontaneous generation of dislocations within polyethylene lamellae sufficient to cause coarse crystallographic slip, involving the translation of blocks of material within the crystal phase, only occur for compression ratios greater than three [32], which is far greater than those considered for this work.

There is much prior research on the sub-shock compression of SCPE under various deformation modes; one common method of deformation in both experiments and simulations is isothermal uniaxial compression—also called unconfined compression—where the system is deformed along one axis, labelled *z*, while the *x* and *y* axes have a constant stress condition, which allows them to expand according to the Poisson’s ratio of the material [21]. In contrast, shock simulations typically consider confined compression under a uniaxial Hugoniostat that keeps dimensions transverse to the compression at a fixed length. This is done to isolate the study to a 1D propagating shock wave in the *z*-direction, which avoids complications relating to the nonlinear superposition of shock waves [1,33]. When the transverse (*x* and *y*) lengths are kept fixed, the total system pressure naturally increases to a much greater level than when the transverse stresses are controlled at some small value, e.g., atmospheric or vacuum pressure.

Several studies involving unconfined compression have been used to identify deformation mechanisms as functions of strain rate. Kazmierczak et al. studied the mechanisms of plastic deformation of polyethylene crystals for strain rates of 5.5 × 10^−5^, 1.1 × 10^−3^, and 5.5 × 10^−3^ s^−1^, and different crystal thicknesses [34]. For uniaxial compression of HDPE, the relationship between true stress and strain rate was shown to follow a logarithmic dependence for a wide range of strain rates between 10^−4^ and 2.6 × 10^3^ s^−1^ [35]. Furthermore, Brown et al. show that the relationship between true stress and temperature follows a linear trend [35]. Kim et al. simulated SCPE models under unconfined compression at two different strain rates, 5 × 10^6^ s^−1^ and 5 × 10^7^ s^−1^ [21]. They found that the crystallographic slip mechanism dominated the deformation response for the slower strain rate. For the faster strain rate, they first observed an increase in stress and then a subsequent crystallographic slip. Jordan et al. examined the behavior of the speed of sound in polyethylene, elastic moduli, unit cell parameters, and other variables, as a function of pressure, using confined compression [36].

In this work, we examine the effect of shock deformation on lamellar stacks of semicrystalline polyethylene with realistic topological distributions in the noncrystalline regions. Uniaxial Hugoniostatted (*NP_zz_*Hug) equilibrium molecular dynamics simulations are used to sample state points along the Hugoniot curve for shock pressures up to 10 GPa. From these state points, measures of orientational and nematic order are obtained. The evolution of density and stress profiles during the transient equilibration period are also examined. Changes in potential energy as a result of shock are analyzed according to the contributions from the different terms of the potential. From such analyses, we propose some mechanistic interpretations for the storage and dissipation of shock wave energy in a prototypical semicrystalline polyethylene lamellar stack model.

## 2. Materials and Methods

### 2.1. Model Generation

The united atom (UA) force field used in this work was adapted from the original Transferable Potential for Phase Equilibria (TraPPE-UA) [37] by including a harmonic bond potential, as in Bolton et al. [38]. The TraPPE-UA potential was parameterized to capture realistic behavior of vapor–liquid coexistence curves as well as densities at pressures of several hundred MPa [37].

Following the work of Lee et al. [20], semicrystalline polyethylene systems were generated using the Interface Monte Carlo (IMC) method [16,19]. Building and pre-equilibration of the PE systems were conducted using the Enhanced Monte Carlo (EMC) software (version 9.3.4) [39], which has been shown to realistically simulate the crystalline and noncrystalline (i.e., amorphous plus interphase) domains of semicrystalline polyethylene [16,21]. Following the procedures of Ranganathan et al. [25] and Kumar et al. [24], all systems were generated in EMC by first creating a fully crystalline system of 4 × 6 × 112 (*a* × *b* × *c*) orthorhombic PE unit cells. For the fully crystalline system (*χ^c^* = 1.0), henceforth referred to as crystalline polyethylene (CPE), the *a*, *b*, and *c* axes of the orthorhombic unit cells were aligned with the *x*, *y*, and *z* Cartesian axes, respectively. *χ^c^* is the mass-weighted crystallinity fraction as defined in Section 2.5.2. The semicrystalline polyethylene systems with mean, pre-shock values of *χ^c^* equal to approximately 0.44 and 0.81, are henceforth referred to as SCPE44 and SCPE81, respectively. The crystal unit cells were oriented such that the {201} Miller plane was perpendicular to the *z*-axis, where the crystal–amorphous interface is eventually formed; experimental studies by Bassett et al. determined the mean angle between crystalline chains and the normal vector to the crystalline–amorphous interface to be 35°, approximately corresponding with the {201} facet [40]. Subsequent computational studies also showed that this facet resulted in the lowest interfacial energy [16,41]. Next, central layers of amorphous-like density, 72 and 35 unit cells thick between fixed crystals, were created by cutting UA sites from each of the 16 chains, for a total of 2265 and 1046 methylene sites removed from SCPE44 and SCPE81, respectively. The 32 methylene sites at the end of each cut were replaced with methyl sites for both systems. The central layer was then amorphized using 10,000 cycles of both local and global Monte Carlo moves at 10,000 K. A set of MC moves was chosen that preserves the number of tails and the sum of loop and bridge segments while changing the overall topology of segments in the amorphous domain (the *NN_e_VT* ensemble, where *N_e_* is the number of methyl sites) [16]. This step was followed by a step-wise cooling sequence at temperatures of 10,000, 5000, 2000, 1000, 750, 500, 400, and 300 K, each step lasting for 20,000 Monte Carlo (MC) simulation cycles. Ten independent configurations for each SCPE system were generated in this way.

After generation, SCPE44 and SCPE81 were simulated using molecular dynamics (MD) in the canonical (*NVT*) ensemble for 2 ps to stabilize the temperature at approximately 300 K. For CPE, one perfectly crystalline configuration was created and 10 different trajectories were initiated by assigning velocity distributions at 300 K with different starting seeds and allowing each to equilibrate under isothermal–isobaric (*NPT*) conditions. All molecular dynamics (MD) simulations were conducted using the LAMMPS software package [42,43] and thermalized throughout both crystalline and noncrystalline layers by MD in either the *NVT* or *NPT* ensemble. The time step of integration was 2 fs. To control pressure and temperature, respectively, the barostat and thermostat methods implemented in LAMMPS follow the form of Shinoda et al. [44], which combines the Nosé–Hoover and Parrinello–Rahman methods; the pressure damping parameter was 2000 fs and the temperature damping parameter was 200 fs. Equilibration was confirmed by ensuring that the thermodynamic parameters (total energy, enthalpy, pressure, and density) of the system fluctuated about the mean values with negligible drift over a period of at least 10 ns. The deviation from the mean was measured by calculating the coefficient of determination, *r*^2^, for the thermodynamic parameters vs. time; if this value is small (<0.01 for this work), then the deviation of the trend from its mean value is better explained by random fluctuations rather than any change in the mean value itself.

### 2.2. Shock Simulation

Following equilibration in the unshocked state (pressure *P* = 0 GPa), the systems were then re-equilibrated to a new state consistent with uniaxial shock using the *NP_zz_*Hug method of Ravelo et al. [8], which is an equilibrium Hugoniostat method that drives the system to a new equilibrium state consistent with the RH conditions. Compression was limited to the lamellar stack direction for the SCPE models, and the crystallographic chain direction for the CPE model. Lateral dimensions were held at fixed length. The method approaches the Hugoniot state by adjusting the equations of motion in a manner similar to the Nosé–Hoover barostat and thermostat, such that the system pressure and energy oppose deviations from values prescribed by the RH conditions. To avoid complications due to bond breaking and the more intricate reactive force fields required to describe them, 10 GPa was chosen as the upper limit of shock pressures, at or below which chemical reactivity is insignificant in real polyethylene systems (c.f. [28]). The Hugoniot state up to 10 GPa was also validated against Hugoniot curve data from both experiments and density functional theory simulations [28,45,46]. Each Hugoniostat simulation was carried out in seven subsequent levels of 11 output steps each; at each level, *k*, data were output every 10*^k^* time steps for 0 ≤ *k* ≤ 6. This was done in order to probe long-term behavior while also focusing on trends that may occur at intermediate and short time scales. Averages reported henceforth for each system either consider the final equilibrium state of the Hugoniostat simulation or are temporal averages at intermediate points during extended Hugoniostat trajectories after equilibration. The pressure and temperature damping parameters for the Hugoniostat simulations were the same as those used for NPT simulations.

For Hugoniostat shock simulations in which the *z*-axis is compressed while the *x*- and *y*-axes are held at constant length, symmetry prevents any significant transverse slip mechanisms, e.g., (100)[010]. According to Bartczak and Galeski, coarse crystallographic slip is generally caused by the heterogeneous nucleation of dislocations [31]; one should note that, in this somewhat idealized computational model, there are relatively few crystal defects that would encourage such a coarse slip. The only ones that may occur are methyl groups that moved into the crystalline region near the lamellar interface.

### 2.3. All-Atom Models

To check the validity of simulations conducted using the TraPPE-UA force field in select situations, a few representative configurations were converted to all-atom (AA) representations and modeled using the OPLS-AA force field [47]. Because of the considerably larger computational and memory costs of the AA models compared to the UA models, only one configuration for each system type was converted and then run using the Hugoniostat.

To convert a UA model to AA, each UA site was first converted to either a methylene or methyl carbon. Next, explicit hydrogens were inserted using geometric criteria based on the local configuration of the alkane chain, in a manner similar to the reverse-mapping procedure described by Brayton et al. [48]. Newly formed angles and dihedrals were identified based on the bond connectivity of the AA representation of the chain. Then, the potential energy was minimized, followed by MD simulation using the OPLS-AA force field with a timestep of 1 fs for 1 ps in an NVT ensemble to stabilize the temperature. Finally, the output data file from the NVT run was used as input for the Hugoniostat simulations.

### 2.4. Order Parameters

Using position and velocity information from the MD trajectory, three order parameters are calculated on a per-UA basis. These are the nematic order parameter, *p_2_*, the orientational order parameter, *S_z_*, and the specific volume, *v*. Here, the nematic order describes the degree of coalignment of nearby bond chords with a reference bond chord within a local region of space, whereas the orientational order describes the degree of alignment of each bond chord with a reference direction, in this case the direction in which shock pressure is applied. Specifically, the *p_2_* order parameter for atom *i* was calculated using [49]

(4) $$p_{2,i}=\frac{3}{2}{\langle \cos^{2}\theta_{ij}\rangle }_{j}-\frac{1}{2},$$

where *i* is the index of the bond chord from atom *i* − 1 to atom *i* + 1 within the chain under consideration, and *j* indexes the neighboring chords within a cutoff radius *r_ij_* < *r_p2_*, here taken to be 1 nm. *p_2_* takes values close to 1 for chords oriented nearly parallel (or antiparallel) to their neighbors, 0 for randomly oriented chords, and −1/2 for chords oriented perpendicular to their neighbors. *S_z_* for atom *i* takes a similar form except that the angle, *ϕ*, is that between the bond chord and the Cartesian unit vector $\hat{z}$:

(5) $$S_{z,i}=\frac{3}{2}\cos^{2}\phi_{i}-\frac{1}{2}.$$

The third order parameter, *v*, is determined via Voronoi tessellation [50], which determines the convex polyhedron surrounding each UA containing the space closer to that UA than any other UA in the system. The specific volume defined on a per-UA basis is then the ratio of the volume occupied by that polyhedron to the mass of the UA. The periodic boundary conditions in the system are accounted for by first replicating the system across each plane of the simulation box (resulting in 3^3^ identical subsystems) and then computing the Voronoi tessellation for this larger system, using the Voronoi polyhedra of the central subsystem for the calculation of specific volumes.

### 2.5. Clustering Analysis

#### 2.5.1. Selection of the Clustering Method

To distinguish trends in the different regions of the system (crystalline vs. noncrystalline), a clustering algorithm was used to segregate the UAs into the two different populations. A clustering algorithm was chosen for this purpose, primarily due to its ability to classify atoms optimally into a finite set of distinct populations. This approach avoids the requirement of selecting, a priori, a threshold value for the classification of sites into one cluster or the other. For example, in prior work we have used a local nematic order parameter (*p_2_*) to classify UAs as crystalline or noncrystalline, with a threshold based on the minimum in the distribution function of *p_2_* for a thermally equilibrated, partially crystallized system [49]. However, under nonequilibrium conditions such as flow, this distribution function changes dynamically, so that the threshold value should change as well [51]. The clustering algorithm avoids this difficulty by defining clusters such that a loss function *L(p_2_)*, defined as the sum of squared distances in the *p_2_* space from each UA to the mean *p_2_* value for the cluster to which it is assigned, is minimal [50,52]. One needs only to specify the number of clusters a priori and provide an initial guess for the mean of each cluster, which can be handled automatically by algorithms such as kmeans++ [53].

Both fuzzy c-means (FCM) [54] and k-means [53,55] were employed for clustering, but it was found that FCM provided more consistent results among different initial configurations, resulting in lower standard errors of several variables as functions of pressure (see Supplementary Materials for extended discussion). FCM assigns to each UA a probability of membership in each cluster (see Section 2.5.2). The improved consistency of FCM makes physical sense because SCPE contains interphase regions where a transition occurs between fully crystalline and fully noncrystalline UAs; FCM can account for the partially crystalline character of sites within the interphase by assigning to each UA a finite probability of being crystalline, with the complementary probability of being noncrystalline, whereas k-means uses a strictly binary classification (i.e., a UA is either crystalline or noncrystalline). Thus, to calculate the mean values of variables not included in the clustering, FCM weights contribute to the mean via membership probability, so that outliers have less influence on the statistic. FCM requires an additional adjustable parameter, *m*, that is the exponent of the fuzzy partition matrix; the exact meaning of this parameter is clarified in Section 2.5.2. Different values of the exponent could be chosen for a particular problem, but in this work reasonable results were obtained using a constant value of 2. This value is consistent with the sum of the squared errors objective function [56].

**Figure 1:**
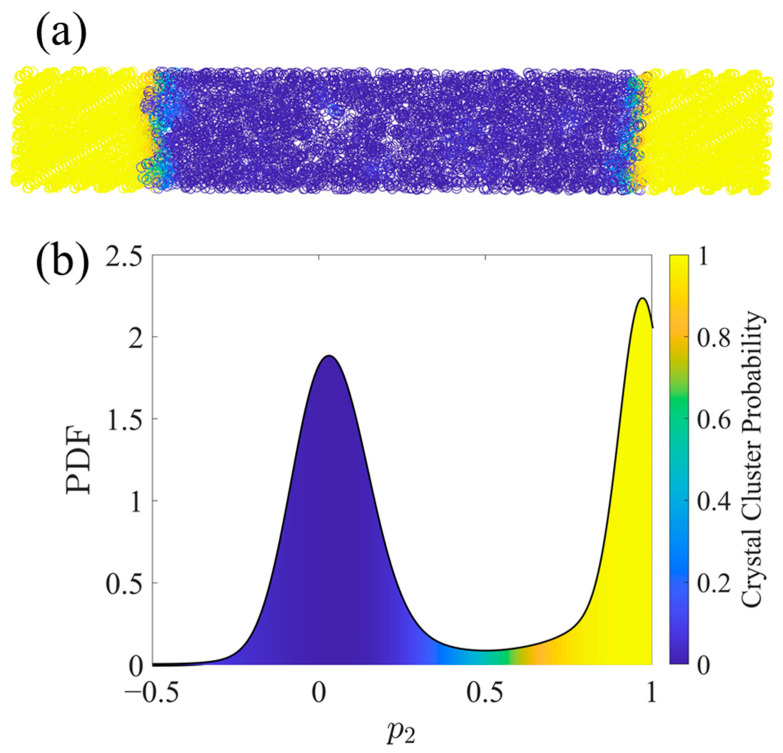
An example of clustering an SCPE44 configuration by the per-atom variable *p_2_* using the FCM algorithm. United atoms are colored by their probability of being included in the crystalline population. (**a**) shows the UAs in Cartesian space while (**b**) shows a probability density function (PDF) of *p_2_* with shading under the curve denoting the crystal cluster probability (*f*_i_^c^) as calculated via FCM. The PDF was smoothed using a kernel density estimate with normal distribution kernel functions [60]. Kernel density estimation is a nonparametric method to estimate a PDF using kernel functions as weights for the contributions from each of the discrete sample points.

In addition to the nematic order parameter, *p_2_*, two other order parameters were considered individually or together for clustering purposes: specific volume, *v*, and orientational order, *S_z_*. Using different combinations of these order parameters in the clustering leads to 2^3^ possible clustering criteria. Silhouette plots [57] were used to evaluate the different combinations, leading to the conclusion that *p_2_* alone provides the best quality clustering. All Silhouette plots used to judge the clustering quality are shown in the Supplementary Materials. Figure 1 illustrates a typical result of this clustering method for an SCPE system. Importantly, clustering in this way has no explicit dependence on the UAs’ positions in Cartesian space and in fact does not guarantee spatial contiguity. However, by definition, a UA with a *p_2_* value near 1 must be in a local neighborhood with consistent alignment of its bond chords, so spatial segregation typically accompanies nematic order segregation, as is depicted in Figure 1.

Before clustering with the above variables, they were converted to *Z*-scores on a per-variable basis. In other words, the data were centered using their mean value and then scaled using the inverse of their standard deviation. This was carried out because the clustering algorithms operate by minimizing the sum of squared distances from each observation to the centroid of its cluster (the mean position of all members within the cluster); if the data were given in different units, the weighting of that variable would be affected. *Z*-scores, on the other hand, are dimensionless and weight each variable roughly equally. For a different application, it may be desirable to control the weighting of each variable, in which case a different scaling may be used.

#### 2.5.2. Statistics of Order Parameters Using Clustering

FCM assigns a probability *f_i_^k^* that the *i*th UA belongs to the *k*th cluster for all *i* ∈ {1, …, *n_i_*} and *k* ∈ {1, …, *n_k_*}. $\sum_{k}f_{i}^{k}=1$ for all *i*, so that the *i*th UA must be fully accounted for in the defined clusters. The centroid of each cluster is denoted by *C^k^*. *C^k^* and *f_i_^k^* are defined by the following simultaneous equations [56]:

(6) $$f_{i}^{k}={\left[\sum_{j=1}^{n_{k}}{\left(\frac{{\left\|x_{i}-C_{k}\right\|}^{2}}{{\left\|x_{i}-C_{j}\right\|}^{2}}\right)}^{1/(m-1)}\right]}^{-1}$$

(7) $$C_{k}=\frac{\sum_{i=1}^{n_{i}}{\left(f_{i}^{k}\right)}^{m}x_{i}}{\sum_{i=1}^{n_{i}}{\left(f_{i}^{k}\right)}^{m}},$$

where *m* is the exponent of the fuzzy partition matrix (*m* = 2 in this work) and *x_i_* is the datum for the *i*th UA used for clustering. The dimensionalities of both *C^k^* and *x_i_* are equal to the number of variables used for clustering. For any order parameter *q_i_* assignable to UA *i*, the mean value of that order parameter for each cluster *k* is defined as

(8) $$\left\langle q^{k}\right\rangle =\frac{\sum_{i}f_{i}^{k}q_{i}}{\sum_{i}f_{i}^{k}}.$$

Equation (8) is used in this work to calculate averages separately for crystalline and noncrystalline populations of SCPE systems. The probability assignments to each UA can also be used to define a cluster fraction, *χ^k^*, representing the contribution of the cluster *k* to the entire system. The cluster fraction is defined as

(9) $$\chi^{k}=\frac{1}{n_{i}}\sum_{i}f_{i}^{k}.$$

Note that, in the UA representation of the PE systems, all methylene UAs have the same mass. Thus, the definition in Equation (9) is essentially the mass-weighted cluster fraction of the system.

In certain contexts, especially when comparing computational and experimental methods of partitioning systems into distinct populations, it is desirable to calculate a cluster fraction that is weighted by a specific order parameter, *q*. Different experimental techniques may naturally measure crystallinity in terms of volume fraction (e.g., with Raman scattering [58]) or other intensive properties [59]. The output of a clustering algorithm also provides a means to compute such parameter-weighted cluster fractions as

(10) $$\chi_{q}^{k}=\frac{\sum_{i}f_{i}^{k}q_{i}}{\sum_{i}q_{i}}$$

for a specific order parameter, *q*, and any cluster, *k*. Note that the definitions of cluster fractions in Equations (9) and (10) have the property that $\sum_{k}\chi_{q}^{k}=1$. Henceforth, for *n_k_* = 2, the superscript “*c*” denotes the crystalline cluster while the superscript “*nc*” denotes the noncrystalline cluster.

## 3. Results and Discussion

### 3.1. Hugoniot Post-Shock States

#### 3.1.1. Pressure versus Specific Volume

**Figure 2:**
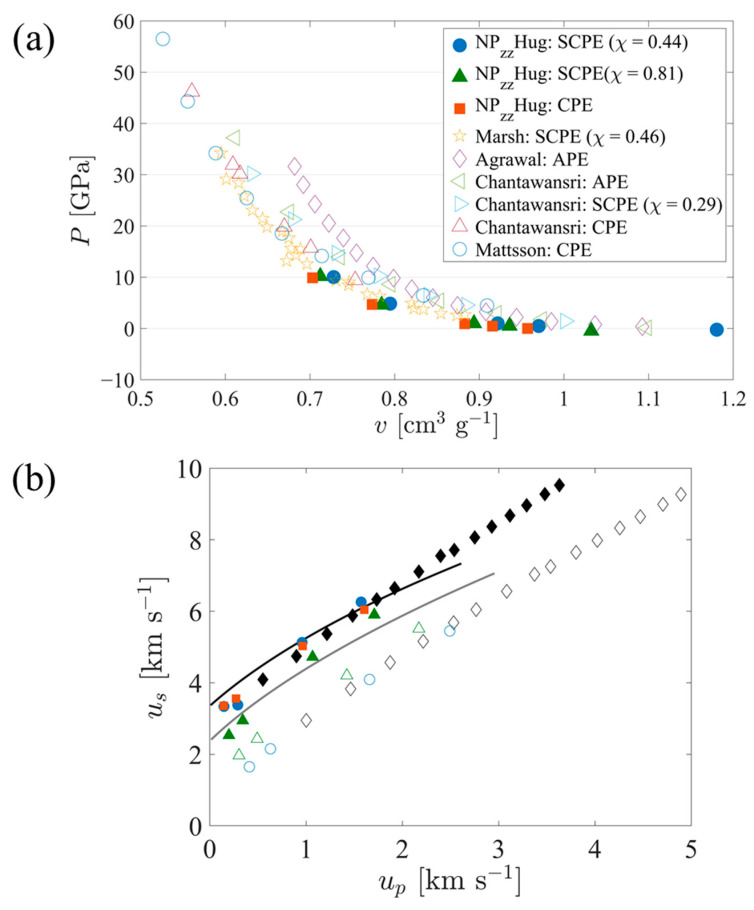
(**a**) Hugoniot *P*-*v* curves for SCPE models compared to experimental data and other simulations results reported in the literature. Values for SCPE (*χ^c^* = 0.44 and 0.81) and CPE are from the current work (filled symbols). Experimental values (gold stars) were obtained from the LASL Shock Handbook [46], where data are reported for experiments in which an explosively driven flying plate was used to induce shock waves in bulk polyethylene (*ρ_0_* = 0.916 g/cm^3^). MSST molecular dynamics simulation data of an AA model of amorphous PE (magenta diamonds) come from Agrawal et al. [64]. DFT simulation data for three different crystallinities of PE (triangles) come from Chantawansri et al. [45]. DFT-AM05 temperature ramp simulation data of CPE (blue circles) come from Mattsson et al. [28]. (**b**) Hugoniot *u_s_* vs. *u_p_* curves for SCPE models compared with results reported in the literature. Values for SCPE44 (blue circles), SCPE81 (green triangles), and CPE (orange squares) are from the current work. Simulation data used for comparison come from Chantawansri et al. [45] (black diamonds). For all of the simulation data, filled symbols indicate CPE (or crystalline regions, in the case of semicrystalline models) while empty symbols indicate APE (or noncrystalline regions, in the case of semicrystalline models). Data are also compared with the theoretical curves of Pastine [62] for CPE (black line) and APE (grey line).

To validate the models and simulations used in this work over the pressure range of interest, Hugoniot curves were constructed using the equilibrated states of systems shocked to different pressures. Figure 2a shows a comparison of these curves in *P-v* space to experimental data and other simulation results previously reported in the literature for polyethylene. The CPE data in Figure 2a are essentially the same as those previously reported in Hsieh et al. [61]. The results obtained in this work are quite close to the experimental trend reported by Marsh [46]. The results of MD simulations by Agrawal et al. [48] deviate the most from experimental data, behavior which they attribute to a low initial density. However, Agrawal et al. noted that scaling all specific volumes by their respective ambient or zero-pressure values brought their data into accord with the theoretical trends of Pastine [62].

The work of Chantawansri et al. also presented results for a model of semicrystalline polyethylene [45]; they used a simplified “layered” structure that fused together purely crystalline and purely amorphous PE (APE) chains such that the crystallographic *c*-axis was perpendicular to the interface between the two regions. They observed larger shifts in the curves of pressure vs. volume with increased crystallinity compared to that observed here. To provide a closer look at the effect of crystallinity, Figure 2b compares the simulation data obtained in this work, converted to *u_s_-u_p_* space using Equations (1)–(3), with the simulation data from Chantawansri [45] as well as the theoretical curves for purely amorphous and purely crystalline PE from Pastine [62]. For crystalline and noncrystalline regions of SCPE simulations, the conversion is applied using the mean specific volumes of the corresponding clusters. For CPE and the crystalline regions of semicrystalline models, all three sets of data are fairly consistent for *u_p_* approximately equal to or exceeding 1 km/s. SCPE81 shows a lower shock speed than the other data sources for lesser values of *u_p_*. For APE and the noncrystalline populations of the current semicrystalline models, the simulation data from Chantawansri et al. and the current work are fairly consistent, while the Pastine curve shows greater values of *u_s_*. Plots of *u_s_* vs. *u_p_* tend to decrease emphasis on the initial density of the system because both speeds are linearly proportional to the square root of initial density [8]; trends in *u_s_* vs. *u_p_* are empirically known to often follow a linear relationship [63], although the intercept of the relationship does also scale with the square root of initial density.

#### 3.1.2. Temperature

**Figure 3:**
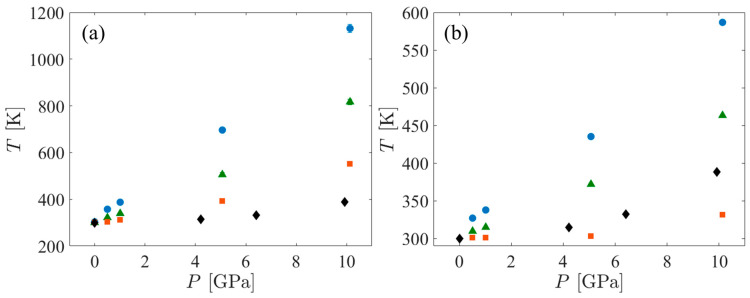
*T* vs. *P* for the SCPE44 (blue circles), SCPE81 (green triangles), and CPE (orange squares) systems. Also shown are the *T* vs. *P* data for polyethylene from DFT-AM05 temperature ramp simulations conducted by Mattsson et al. [28] (black diamonds). (**a**) shows the UA data while (**b**) shows the AA data.

**Figure 4:**
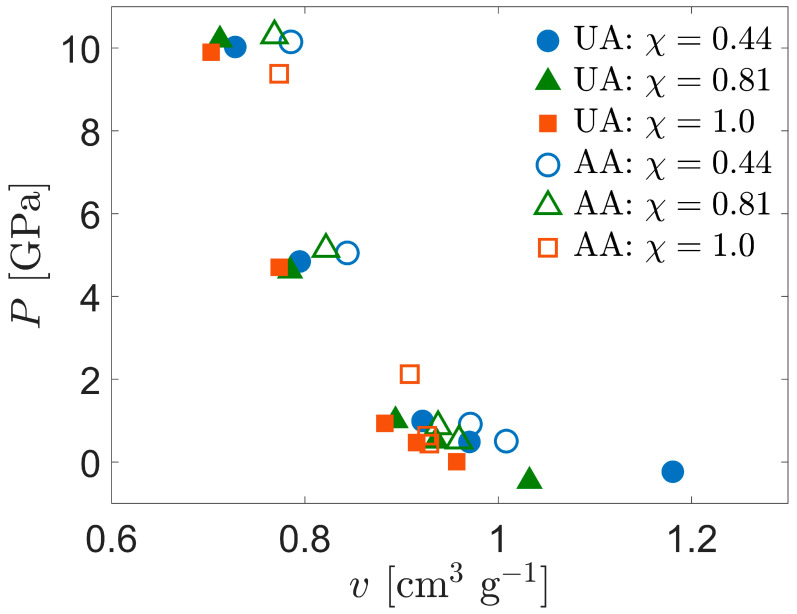
Comparison of Hugoniot *P*-*v* curves for UA and AA models of SCPE.

The temperature increase associated with the application of shock pressure depends on the heat capacity of the system. Heat capacity tends to be strongly model-dependent. Molecular dynamics simulations of fully flexible AA models tend to overestimate heat capacity because they treat all degrees of freedom classically, including those associated with high frequency vibrations that would be more properly considered as quantum mechanical in nature [65]. On the other hand, UA models tend to underestimate heat capacity because they eliminate numerous vibrational degrees of freedom, including some that would be activated at the temperatures experienced by the system. Thus, the temperature of the UA systems under shock always increases more than that of the AA systems. To bracket the actual temperature increase, a few Hugoniostat simulations using the AA force field were performed. The variation of temperature with shock pressure is shown in Figure 3. When comparing the temperature increases in SCPE44 and SCPE81 as functions of the shock pressure, the UA systems increase by approximately twice as much as the corresponding AA systems. However, this trend was not observed for the CPE systems. The temperature increase in the AA systems is very small for all pressures up to approximately 10 GPa, where it is approximately 30 K. The corresponding UA system increased by approximately 260 K, or approximately 8.6 times the AA pressure increase. Along a Hugoniot curve,

(11) $${\left(\frac{\partial T}{\partial P}\right)}_{Hug}={\left(\frac{\partial T}{\partial v}\right)}_{Hug}{\left(\frac{\partial v}{\partial P}\right)}_{Hug},$$

where *v* is the specific volume. From Figure 4, the compressibility along the Hugoniot curve (${\left(\partial v/\partial P\right)}_{Hug}$) is only about 10% different between the UA and AA systems, clearly not enough to compensate for the temperature difference. We hypothesize that the ratio of constant-pressure to constant-volume heat capacities is closer to unity in the AA CPE than it is for the other systems, so the temperature increase in this system along the Hugoniot curve is correspondingly reduced. The basis for this hypothesis lies in consideration of a much simpler system—an ideal gas heated adiabatically. Although this simpler system is unsuitable for quantitative comparison with CPE in the current work, the temperature increase in CPE may be strongly dependent on the heat capacity ratio, by analogy to the ideal gas.

#### 3.1.3. Orientational Order Parameter

The orientational order parameter, *S_z_*, is used to track the shift in orientation of the bond chords in the crystalline domain with respect to the *z*-axis, which is both the direction in which the shock compression is applied and the direction normal to the interfaces between the crystalline and noncrystalline regions in the lamellar stack. Changes in values of the mean orientational order parameter for the crystalline domains, ⟨*S_z_^c^*⟩, are potentially indicators of crystallographic slip + compression in the SCPE systems, necessitated by the geometric confinement placed on the bond chords due to the compression. It is important to note that UA models tend to underestimate the energy barrier preventing crystallographic slip; see, e.g., Olsson et al. [66]. Thus, while configurational changes to the UA PE systems due to crystallographic slip are realistic, it is possible that the role of crystallographic slip may be overemphasized in these systems relative to other deformation mechanisms that would be more prominent in a more detailed atomistic model.

**Figure 5:**
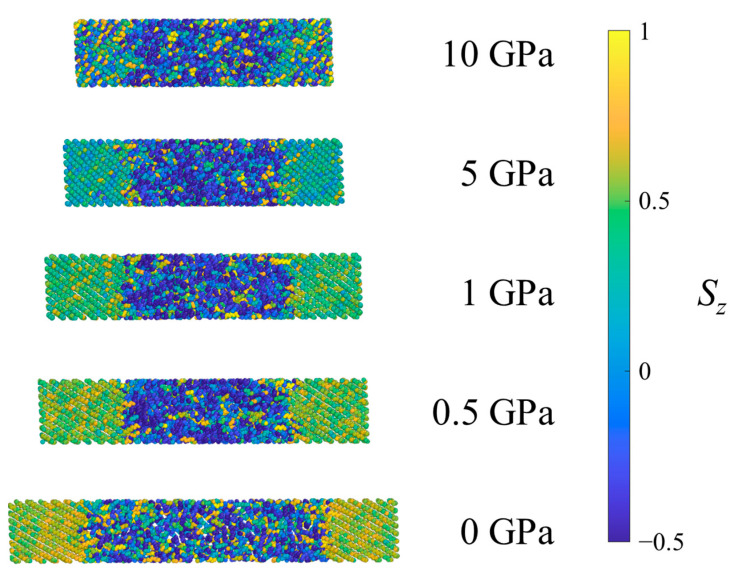
Example SCPE44 system under ambient conditions (0 GPa) and after equilibration at several pressures in Hugoniostatted shock simulations. Atoms are colored according to the orientational parameter *S_z_*.

Figure 5 shows all of the methylene UAs in an SCPE44 system colored according to the *S_z_* value of the associated bond chord. Methyl UAs are not shown because they cannot be assigned a bond chord in the same way. At low pressures, as the shock pressure increases, bond chords in the crystalline region tilt uniformly away from the *z*-axis, so their *S_z_* values decrease on average. At the highest pressure (10 GPa) the crystalline clusters begin to exhibit greater variance in *S_z_*, an indication of the decrease in crystallographic order.

**Figure 6:**
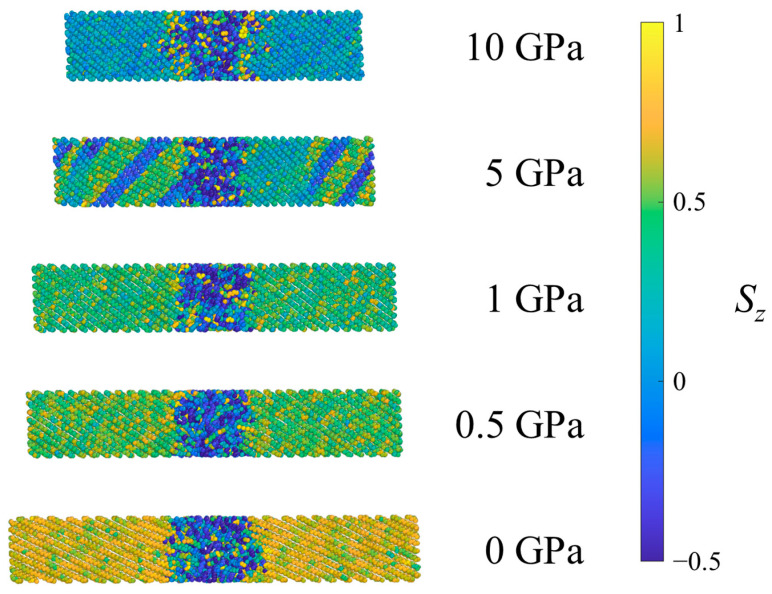
Example SCPE81 system under ambient conditions (0 GPa) and after equilibration at several pressures in Hugoniostatted shock simulations. Atoms are colored according to the orientational parameter *S_z_*.

Figure 6 shows the UAs of one system of SCPE81 colored according to the *S_z_* for different pressures. Interestingly, kink band formation is observed in SCPE81 at 5 GPa for this configurational seed. A kink band forms when crystallographic slip is inadequate to accommodate compression within a crystal uniformly, so that a section of the crystal rotates cooperatively, localizing the deformation [31]. For different configurational seeds (not shown), kink bands occur in different locations and with different numbers of bands. Kink bands with widths of roughly 10 bond lengths across were observed in 7 out of 10 configurational seeds at 5 GPa, while the remaining seeds exhibited shorter crystalline defects roughly 2 bond lengths across. This kink band formation in the crystalline structure is identified as another feature of shock energy absorption that appears under special conditions. For *P* < 5 GPa, the bond chords tilt uniformly, and thus crystallographic slip + compression is the dominant mechanism at low shock pressures. At 10 GPa, the combination of the decreased nematic order of the crystal and higher temperature apparently decrease the barrier to tilt and disrupt the organized formation of large kink bands; only 2 out of 10 configurational seeds exhibit kink bands with widths of roughly ten bond lengths across while the remaining seeds exhibit shorter crystalline defects roughly two bond lengths across. Thus, prominent kink bands are observed mainly at intermediate shock pressures and in systems with sufficiently thick crystalline lamellae.

**Figure 7:**
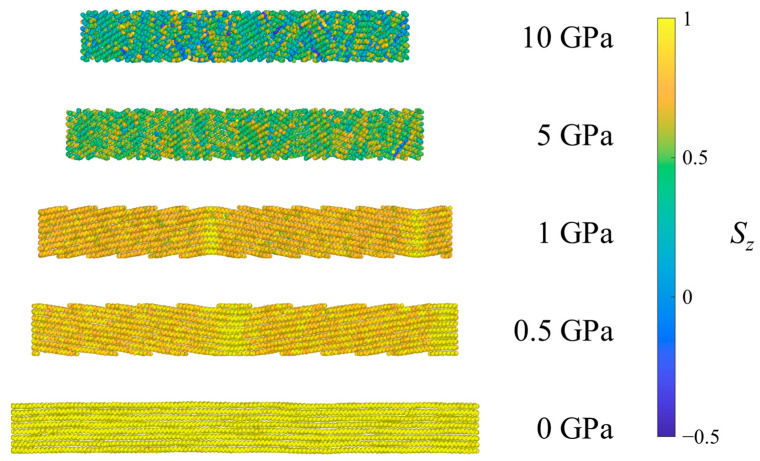
Example CPE system under ambient conditions (0 GPa) and after equilibration at several pressures in Hugoniostatted shock simulations. Atoms are colored according to the orientational parameter *S_z_*.

Figure 7 shows the UAs of one system of CPE colored according to the *S_z_* for different pressures. An important observation is that the CPE system, due to constraints on the system geometry, is not free to tilt by large angles as the crystalline regions of SCPE systems are. In CPE, UAs on each side of the periodic boundaries perpendicular to the *z*-direction must be bonded, so tilting in one direction must be accompanied by tilting in the opposite direction elsewhere in the crystal and the formation of kink bands, such that the long period of deformation is commensurate with the simulation cell size. Such kink band formation is a typical case of buckling in response to compression. For low pressures, the systems have only two bends separating regions of tilt by different angles but similar *S_z_* values. At 5 GPa, the long period or wavelength of buckling is reduced, resulting in the formation of multiple kink bands and higher tilt angles. Finally, at 10 GPa most of the nematic order in the crystal has diminished and the system loses long-range spatial correlations, forming numerous small regions of different alignment. Thus, for fully crystalline systems, kink bands are observed at all shock pressures, but the long period decreases with increasing pressure and eventually breaks up into disordered domains at the highest shock pressure, analogous to the large crystalline domain in SCPE81.

**Figure 8:**
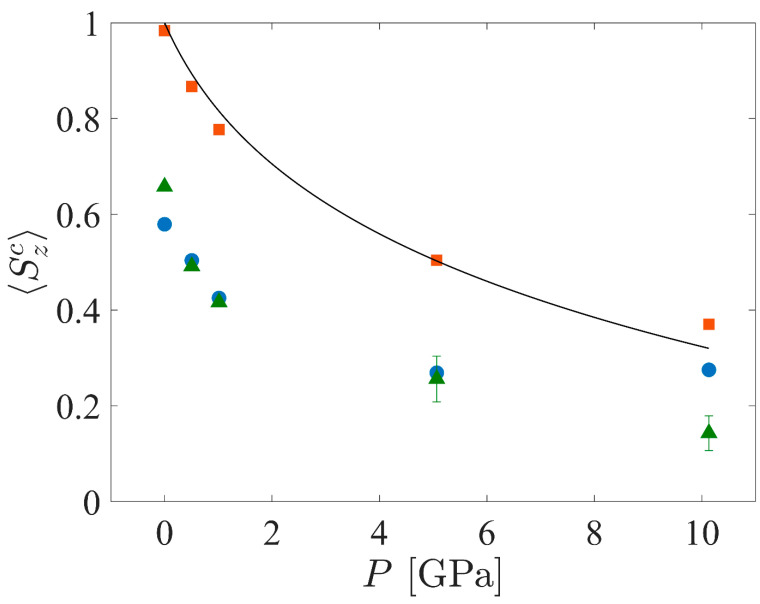
Mean orientational parameter, ⟨*S_z_^c^*⟩, vs. *P* for the crystalline populations of SCPE44 (blue circles), SCPE81 (green triangles), and CPE (orange squares). Also shown is a theoretical prediction of ⟨*S_z_^c^*⟩ vs. *P* for CPE based on the model of Pastine [62].

Figure 8 shows the mean *S_z_* values (⟨*S_z_^c^*⟩ as defined in Section 2.5.2, Equation (8)) of the crystalline cluster as a function of pressure for the three systems. Through construction, the CPE system at *P* = 0 GPa is almost perfectly aligned with the *z*-axis; however, the bond chords of this system tilt to form kink bands in response to the strain imposed by the compression. Fluctuations of UA coordinates about those of the perfect crystal may dictate the direction that the bond chords tilt—this direction is not always consistent among the different starting seeds, but the absolute value of the angle is fairly consistent. Also shown in Figure 8 is a theoretical prediction of ⟨*S_z_^c^*⟩ vs. *P* for CPE using Pastine’s model [62], assuming that the strain in the crystal manifests entirely as tilt of the bond chords. Assuming that the strain in the crystal is

(12) $$\epsilon^{c}=1-\left|\cos\phi \right|,$$

where *ϕ* is the angle the bond chords make with the *z*-axis, ⟨*S_z_^c^*⟩ is calculated according to Equation (5) and *ϵ^c^*(*P*) is determined from Pastine’s theory. The theoretical prediction and the simulation data have a root-mean-square deviation of approximately 0.032; deviations between the data and model may be due to crystallographic strain caused by an excess compression in the *a* or *b* unit cell dimensions (beyond that caused by chain tilt) or differences between the crystallographic unit cell in the current work and that of Pastine.

Figure 8 also shows the dependence of ⟨*S_z_^c^*⟩ on shock pressure for the crystalline domains of the SCPE systems. The orientational order within the crystalline domains for the two different SCPE systems are in close agreement up to 5 GPa but deviate at 10 GPa. The values for SCPE44 exhibit an upward shift after 5 GPa, while the values for SCPE81 appear to be “noisy” for 5 and 10 GPa. The non-monotonic decrease in the ⟨*S_z_^c^*⟩ values with increasing pressure for SCPE44 systems must be a result of interactions with the noncrystalline population because neither the theoretical nor simulation data for CPE show such features. An interchange of strain between the crystalline and noncrystalline regions is not likely because it would be reflected as non-monotonicity in *v* vs. *P* in Figure 2a, for example. Rather, it seems that, for *P* ≈ 10 GPa, there is growth of the population of noncrystalline UAs at the expense of crystalline UAs (i.e., “melting”), thus eliminating some of the less crystalline UAs from the crystalline cluster and increasing the average orientation of the crystalline cluster. The kink boundaries observed for SCPE81 in Figure 6 result in the large error bars observed in Figure 8.

#### 3.1.4. Crystallinity

**Figure 9:**
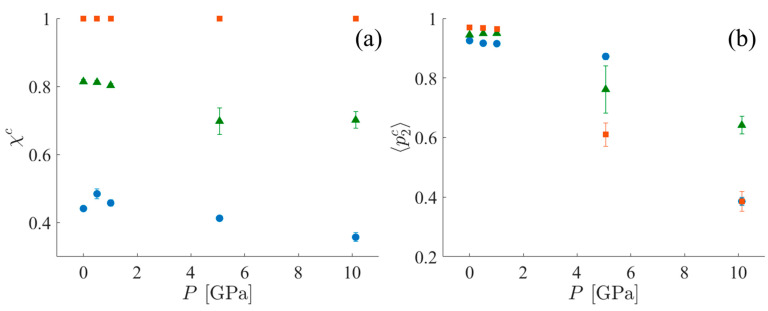
(**a**) *χ^c^* and (**b**) 〈*p_2_^c^*〉 for SCPE44 (blue circles), SCPE81 (green triangles), and CPE (orange squares). Data are averages among the ten different starting configurations with error bars indicating three standard errors.

To characterize the crystallinity of each of the systems, two different metrics are used. The first is the mass fraction of the crystalline population—*χ^c^*, as defined in Section 2.5.2, Equation (9). *χ^c^* alone is insufficient to fully characterize the crystalline order of the system when the crystalline region is imperfect (i.e., without perfect periodicity). For example, this definition always assigns a crystallinity of 100% to the CPE systems because these systems are characterized as a single cluster, even in the presence of kink bands (see the Supplementary Materials for further discussion). However, with increasing shock pressure, the nematic order of the CPE system decreases. Supplementary information is provided by the mean value of the nematic order parameter for the crystalline population, 〈*p_2_^c^*〉, calculated using Equation (8). Trends for both *χ^c^* and 〈*p_2_^c^*〉 in all three systems are shown in Figure 9. Notice that 〈*p_2_^c^*〉 for SCPE81 at 5 GPa is less than the value for SCPE44 due to the formation of kink bands and the loss of the nematic order of UAs in between these kink bands. It appears that the crystallinity, *χ^c^*, changes relatively little under the application of shock, but the nematic order within the crystalline lamellae, 〈*p_2_^c^*〉, decreases significantly.

#### 3.1.5. Potential Energy Contributions

**Figure 10:**
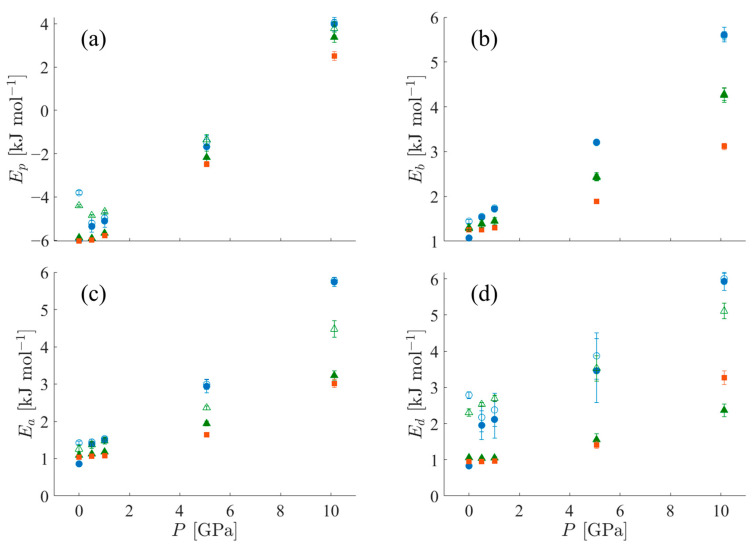
Potential energy contributions per UA for crystalline (filled symbols) and noncrystalline (empty symbols) populations of SCPE44 (blue circles), SCPE81 (green triangles), and CPE (orange squares, no non-crystalline population). The contributions are (**a**) pair (Van der Waals) energy, (**b**) bond energy, (**c**) angle energy, and (**d**) dihedral energy.

The FCM clustering analysis is used to determine the mean potential energy contributions of both the crystalline and noncrystalline clusters of the systems separately. Potential energy contributions for the TraPPE-UA force field include nonbonded (pair) and bonded (bond, angle, and dihedral) contributions; these contributions are shown in Figure 10. The pair contribution, shown in Figure 10a, is lower for crystalline clusters than noncrystalline clusters because the former has a more stable chain packing arrangement. As pressure increases, the mean pair contributions of the crystalline clusters of both SCPE44 and SCPE81 increase nearly to the levels of the mean pair contributions of the noncrystalline clusters, indicative of decreasing order within the crystal cluster. In contrast, the mean bond contributions in Figure 10b are the same for both clusters within a single system. The bond potential is by far the stiffest in the system, so the sensitivity of bond length displacements to the local environment (crystalline or noncrystalline) is negligible. Thus, the bond potential energy increase can mainly be attributed to the increase in temperature that accompanies increasing pressure along the Hugoniot curve. The angle energy, shown in Figure 10c, is the next stiffest mode in the systems. At low pressure, it is the same for both crystalline and noncrystalline clusters, similar to the bond energy. However, at elevated shock conditions it begins to differentiate for crystalline and noncrystalline clusters, indicating a sensitivity to the local environment. The mean angle contribution of the crystal cluster in SCPE81 closely tracks that of CPE, while the mean angle contribution of the crystal cluster in SCPE44 tracks more closely with that of the noncrystalline population for pressures as high as 10 GPa. Finally, the dihedral energy contribution is shown in Figure 10d; like the pair contribution, it is indicative of decreasing crystallographic order, especially for the case with the lowest crystallinity (SCPE44).

### 3.2. Hugoniostat Transient Evolution

**Figure 11:**
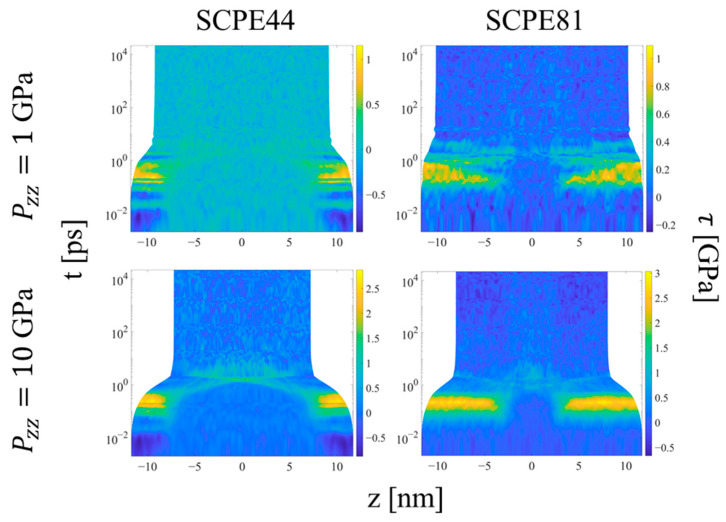
Heat plots for the shear stress as a function of position along the compression direction and time, for two systems and two applied pressures during Hugoniostatted simulations. The color scale is proportional to the shear stress. Plots for all systems and pressures are included in the Supplementary Materials.

**Figure 12:**
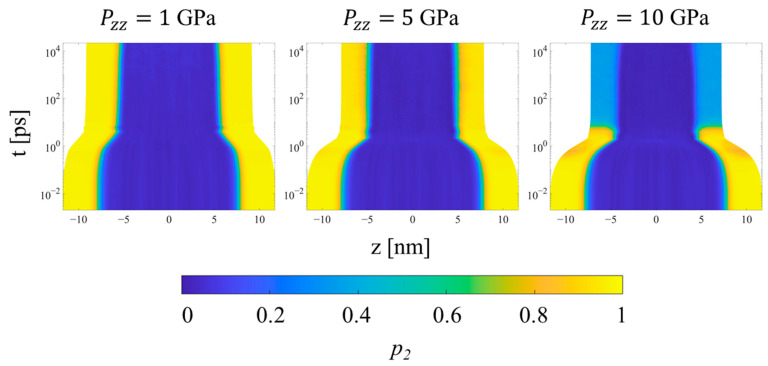
Heat plots of the *p_2_* order parameter for the SCPE44 as a function of position along the compression direction and time, for three different applied pressures in Hugoniostatted simulations. The color scale is proportional to *p_2_*. Plots for all systems and pressures are included in the Supplementary Materials.

**Figure 13:**
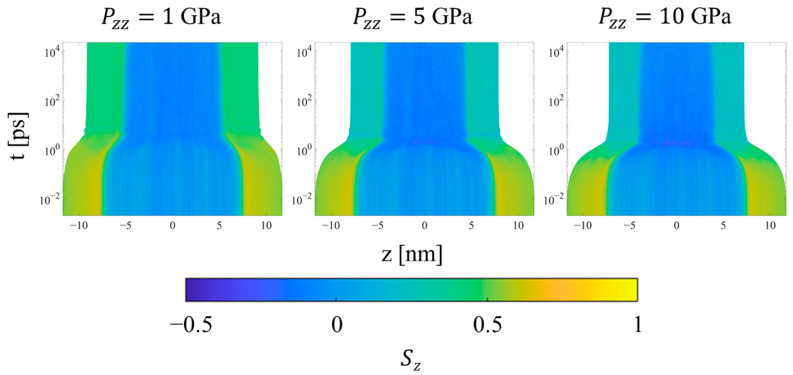
Heat plots of the *S_z_* orientation parameter for SCPE44 as a function of position along the compression direction and time, for three different applied pressures in Hugoniostatted simulations. The color scale is proportional to *S_z_*. Plots for all systems and pressures are included in the Supplementary Materials.

Upon the initial application of the shock pressure, one observes two major regimes of compression, as illustrated by the evolution of local order parameters with logarithmic time in Figure 11, Figure 12 and Figure 13. Figure 11 shows the spatial evolution of stress during the equilibration of shock. In the first, transient regime, which extends from approximately *t* = 0.01 to 10 ps, shear stress builds up in the crystalline domains of the systems, followed by compression of the crystalline and noncrystalline domains. The information computed by LAMMPS is *S_αβ_*, where *S* is the negative of the per-UA stress tensor multiplied by volume, and the subscripts denote the components. To calculate per-UA pressure values, components of *S* are negated and then divided by per-UA volumes, calculated via Voronoi tessellation (see Section 2.4 for details). The shear stress for compression in the *z*-direction is computed as [8]

(13) $$\tau =\frac{1}{2}\left(P_{zz}-\frac{P_{xx}+P_{yy}}{2}\right).$$

After a compression time on the order of picoseconds, the crystalline domains show a rise in shear stress to levels approximately half of the applied shock pressure in the *z*-direction. The low resistance to crystallographic slip in the UA models permits rotation of the chain stems in the crystalline domains in response to the shear stress, so that the compressive load is borne more by the softer nonbonded, intermolecular interactions and less by the stiffer bonded, intramolecular interactions; similar behavior was observed for small extensional strains (< 0.08) of UA SCPE models under isothermal uniaxial compression by Kim et al. [21]. At this point, the system experiences significant strain in both the crystalline and noncrystalline domains in response to the applied compressive stress. This behavior indicates that (1) there is a short delay during which the crystalline domain experiences the buildup of transverse and longitudinal stresses with respect to the shock direction that drive crystallographic slip, followed by (2) compression of the crystalline domain to equalize stress. The noncrystalline domain equalizes the stress in the different directions much more rapidly and thus never experiences a significant shear stress. The rise in shear stress would not be expected for compression of pure APE, due to the fast equalization of its stresses.

Once the final system volume is reached, the shear stress is nearly zero, indicating that the strain response serves to equalize the stress in all directions. The pressure also becomes uniform throughout the system, as a result of equilibration to the post-shock Hugoniot state. Figure 12 shows the local *p_2_* order parameter for several systems. The crystalline and noncrystalline domains remain clearly defined over the entire range of pressure. However, referring to Figure 9b, SCPE44 maintains high nematic order in the crystalline domain up to 5 GPa, although this order decreases dramatically at 10 GPa. SCPE81, on the other hand, exhibits a decrease in nematic order at 5 GPa, but with large variance; we hypothesize that this behavior is a consequence of the formation of kink bands in that system. Meanwhile, the orientational order parameter ⟨*S_z_^c^*⟩ confirms the tilting of chain stems in the crystalline domain away from the direction of applied load, as shown in Figure 11. One exception to this general trend is SCPE44, for which crystalline orientational order increases very slightly at high pressure, from 0.270 at 5 GPa to 0.275 at 10 GPa. This counterintuitive increase in ⟨*S_z_^c^*⟩ can be explained by a decrease in *χ^c^* over the same range of pressure.

**Figure 14:**
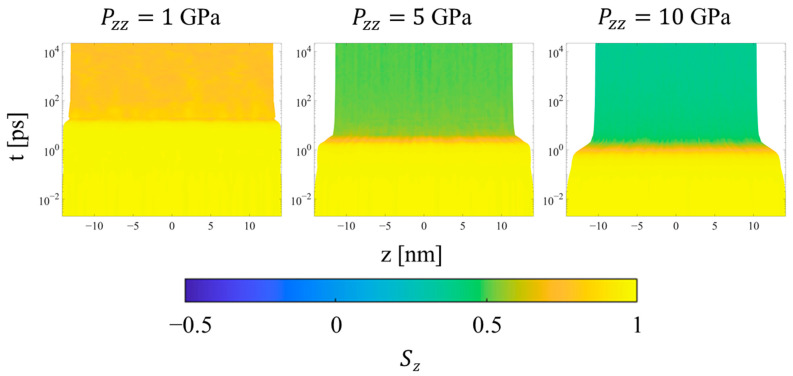
Heat plots of the *S_z_* orientation parameter for CPE as a function of position along the compression direction and time, for three different applied pressures in Hugoniostatted simulations. The color scale is proportional to *S_z_*. Plots for all systems and pressures are included in the Supplementary Materials.

Finally, considering the trends for the CPE system, the uniformity of all metrics throughout the simulation is preserved during all simulations. The density increases uniformly during the transient regime, while the *p_2_* order parameter (not shown) decreases uniformly at higher pressures to values near 0.6 and 0.3 at 5 and 10 GPa, respectively. The orientational order parameter (Figure 14) decreases for all pressures in order to accommodate compression of the crystal region through buckling, kink band formation, and the mechanism of fine crystallographic slip. Interestingly, increasing the applied pressure from 5 to 10 GPa increases the amount of time required for the reorientation to complete, as shown by the intermediate values of *S_z_* during the equilibration regime. In both cases, the decrease in *S_z_* occurs gradually and monotonically even after the final system volume is achieved.

## 4. Conclusions

This work analyzes the shock wave response of model SCPE and CPE systems for the purpose of understanding the changes to the configurational states associated with shocks of different pressures. Shock simulations were conducted using molecular dynamics with an equilibrium Hugoniostat called *NP_zz_*Hug [8]. Clustering based on the FCM algorithm is introduced to allow adaptive clustering in response to changes in the distribution of the nematic order parameter, *p_2_*, so that the trends of individual populations may be analyzed separately. The nematic order parameter is found to distinguish crystalline and noncrystalline UAs within each SCPE system with high fidelity and simplicity. We take advantage of this clustering to focus on the response of the crystalline populations in this work, because they exhibit a variety of deformation mechanisms depending on their initial degree of crystallinity and the applied shock pressure.

Examining the Hugoniostat trajectories, two potential energy storage mechanisms are identified: loss of nematic order within the crystal domain and change in the orientation of the crystal stems with respect to the crystalline–noncrystalline interface via crystallographic slip + compression. Both of these mechanisms increase the potential energy of the system and thus store energy of the shock wave by changing the system configuration. Additionally, for systems of sufficiently high crystallinity (or lamellar thickness), the formation of kink bands is observed within the crystalline region, as evidenced by the data for SCPE81 at 5 GPa in some instances, and for CPE at all shock pressures. The long period of these kink bands decreases with increasing shock pressure, consistent with a increasing energy buckling phenomenon. The formation of kink bands may be more prevalent in experiments than observed here, due to the ease with which crystallographic slip occurs in the UA model. Finally, at the highest pressure (10 GPa), kink bands apparently break up or disappear; we hypothesize that a higher temperature and lower nematic order lead to a decreased energy barrier for local slip and bend formation, and a degree of melting. For CPE, the angle of the chain tilt as a function of shock pressure is well-approximated by Pastine’s theory [62].

At low shock pressures (up to about 1 GPa), all systems exhibit fine crystallographic slip. For the CPE systems, however, this slip is necessarily accompanied by kink band formation because of the lack of compliance imposed by the periodic boundary conditions. For the SCPE systems, on the other hand, the noncrystalline regions act as damping boundary conditions for the crystal chains, allowing them to tilt without kink band formation in response to the development of shear stress. At higher shock pressures, kink bands also begin to form in SCPE81 as further tilt of the crystal chains becomes energetically unfavorable. The SCPE44 systems do not form kink bands at any of the pressures simulated; instead, at 10 GPa and elevated temperature in the Hugoniot state, some of the crystalline population “melts” into the noncrystalline population in order to satisfy the geometric constraints caused by confinement while not altering the tilt angle. This melting is also observed in the convergence of the potential energy contributions of the crystalline populations to those of the noncrystalline populations in SCPE44 at 10 GPa, but not in SCPE81. This is also supported by the lower 〈*p_2_^c^*〉 values in SCPE44 compared to SCPE81 at 10 GPa. In fact, the 〈*p_2_^c^*〉 values in SCPE81 exceed those of the CPE systems at 10 GPa. This behavior suggests that the presence of some noncrystalline material can actually stabilize the crystalline domains against melting in an SCPE system by acting as something of a “shock absorber,” but that too much noncrystalline material (which in this case correlates with lower crystallinity and thinner crystalline domains) can destabilize the crystalline domains with respect to melting. Further study of these systems may be able to more accurately determine an optimal combination of crystallinity and crystalline domain thickness to maintain the integrity of the crystal according to one of the aforementioned metrics.

Fundamentally, the deformation mechanisms observed in all of the simulated PE systems are consequences of the geometric confinement caused by the confined compression of the shock, the temperature increase in the Hugoniot state, and the atomic configuration in the initial state of the system. The modulus for intramolecular compression along the crystallographic *c*-axis (the chain axis) is an order of magnitude greater than the moduli for intermolecular compression along the *a-* or *b*-axes of the unit cell, so most of the deformation occurs through rotation of the unit cell to accommodate compression intermolecularly [62]. If the nematic order of the crystalline regions remains high, then the assumptions used by Pastine’s model hold—namely, that there is at most a linear correction for compression along the *c*-axis and that the spatial arrangement of atoms remains periodic. Thus, we see for shock pressures of < 10 GPa that Pastine’s prediction for stress as a function of strain in CPE can be used to predict the tilt of crystal stems leading to crystallographic slip + compression. These assumptions also approximately hold for the SCPE systems under the same pressure condition, as evidenced by the superposition of the SCPE curves in Figure 8; the downward shift of the SCPE results relative to the CPE result is due to the initial tilt of the crystal stems with respect to the direction of compression. Additionally, the SCPE systems exhibit some melting at high pressure, as indicated by a decrease in *χ^c^* that is not observed for CPE or Pastine’s model. This melting behavior accommodates a portion of the shock energy, as a result of which the nematic order within the crystal clusters does not decay as much for SCPE as it does for CPE.

## Data Availability

Publicly available datasets were analyzed in this study. This data can be found here: https://github.com/jpmikhail/Mechanisms_of_Shock_Dissipation_in_Semicrystalline_Polyethylene.

## Supplementary Materials

The following supporting information can be downloaded at: https://www.mdpi.com/article/10.3390/polym15214262/s1, Hugoniostat Transient Evolution, Figures S1–S3; Evaluation of Clustering Methods by Silhouette Scores, Figures S4–S8. Reference [67] is cited in the Supplementary Materials.
