# Supplementary material for: Mechanisms of Shock Dissipation in Semicrystalline Polyethylene
Source: Polymers (Basel). 2023 Oct 30;15(21):4262. doi: 10.3390/polym15214262 (PMC10650820; doi:10.3390/polym15214262)
Supplement: Supplementary file 1 [file polymers-15-04262-s001.zip › polymers-2666117-supplementary.pdf]

Mechanisms of Shock Dissipation in Semicrystalline Polyethylene – Supplementary Materials

John P. Mikhail, Gregory C. Rutledge*

Department of Chemical Engineering, Massachusetts Institute of Technology, 77 Massachusetts Avenue, Cambridge, Massachusetts 02139, USA

Institute for Solder Nanotechnologies, Massachusetts Institute of Technology, Cambridge, MA 02139, USA

Hugoniostat Transient Evolution

In this section, we present the transient Hugoniostat data for all systems (SCPE44, SCPE81, CPE) and all shock conditions. These data are heat plots of shear stress (τ), the nematic order parameter (p_2), and the orientational order parameter (S_z); all heat plots show the selected variable as a function of elapsed time since the start of the Hugoniostat simulation and the position along the *z*-axis, along which the compression is applied. The data shown are averages among trajectories from the ten different starting configurations, applied in the following procedure: (1) the average length of all systems along the *z*-axis was computed. (2) for each individual system, atomic *z*-coordinates were scaled to relative *z* coordinates ranging from 0 to 1 along the system length. (3) per-UA data from each starting configuration were then binned and averaged into 500 equally-sized intervals along the scaled z-coordinate. (4) these binned data were further averaged among the ten different configurational seeds. (5) the scaled *z*-coordinates for the averaged data were multiplied by the average *z*-length of all systems to display the data in distance units. This

procedure was done independently for each timestep of the simulation. After step (1), it was verified that the systems did not show large deviations in *z*-length among the different starting configurations. The greatest value observed for the standard deviation of *z*-lengths divided by the mean was 1.5% among all systems, pressures, and time points.

Figure S1 shows a heat plot of the shear stress of the systems. The magnitude of the shear stress is only significantly greater than zero for the crystalline regions before they reduce their volume due to the crystallographic slip + compression mechanism. Figure S2 and Figure S3 show heat plots of the nematic and orientational order parameters of the systems, respectively.

Shear Stress

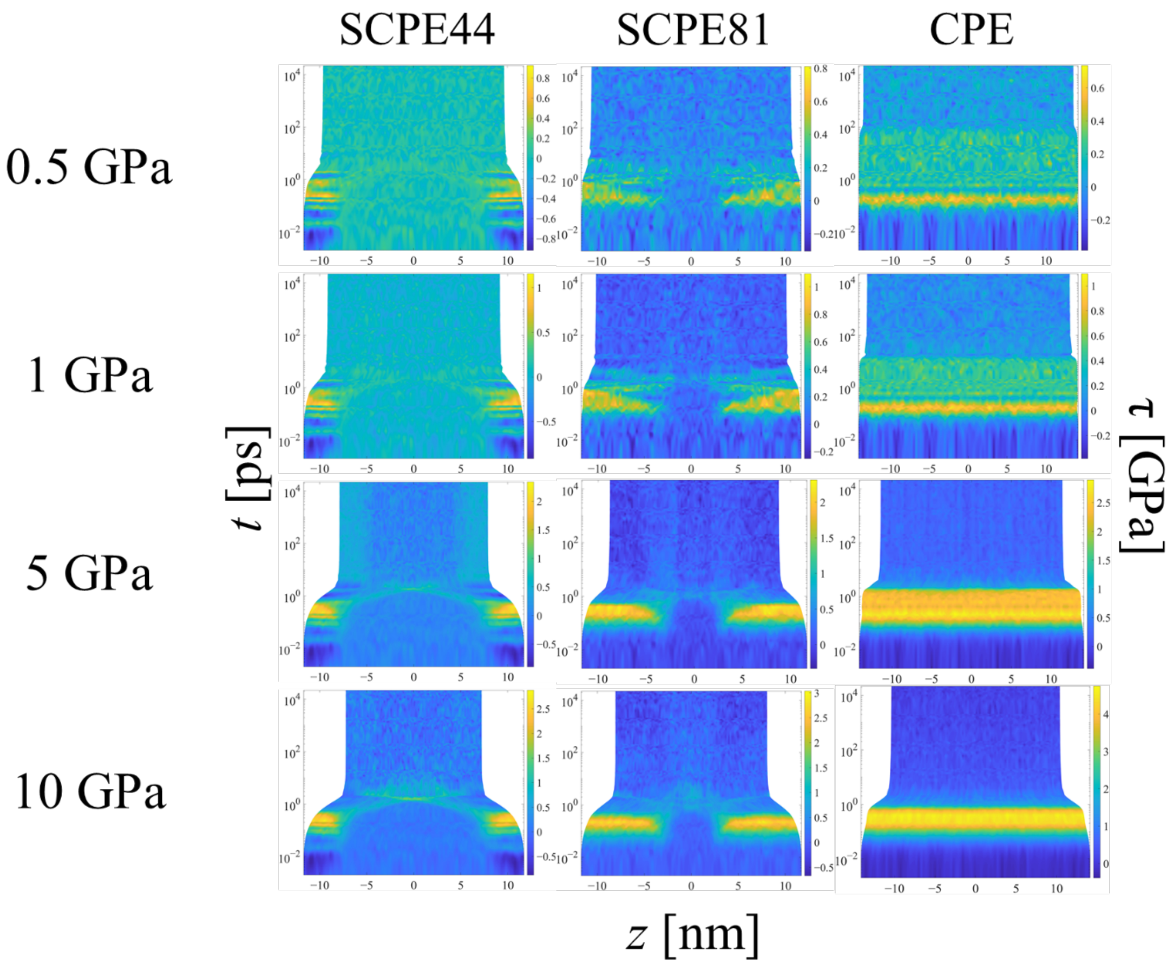

Figure S1. *Contour plots of shear stress, τ, as a function of time and position along the compression axis.*

Nematic Order

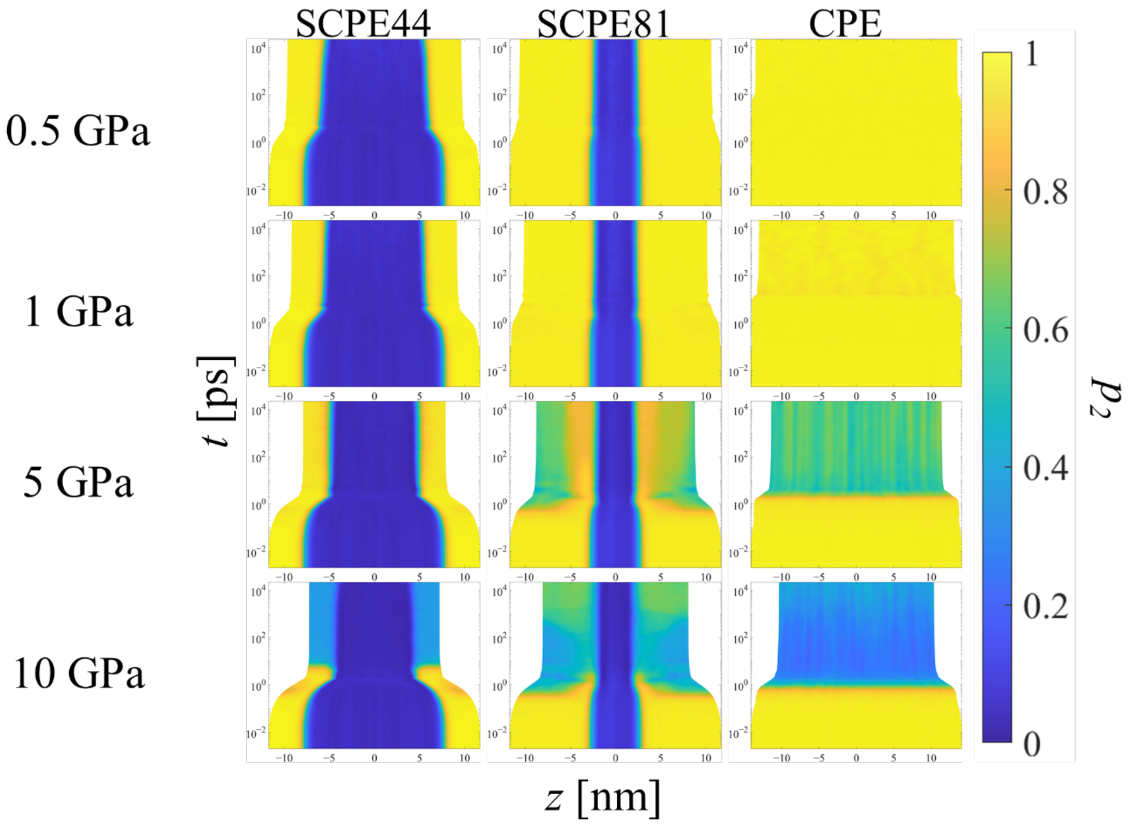

Figure S2. *Contour plots of p_2 as a function of time and position along the compression axis.*

Orientational Order

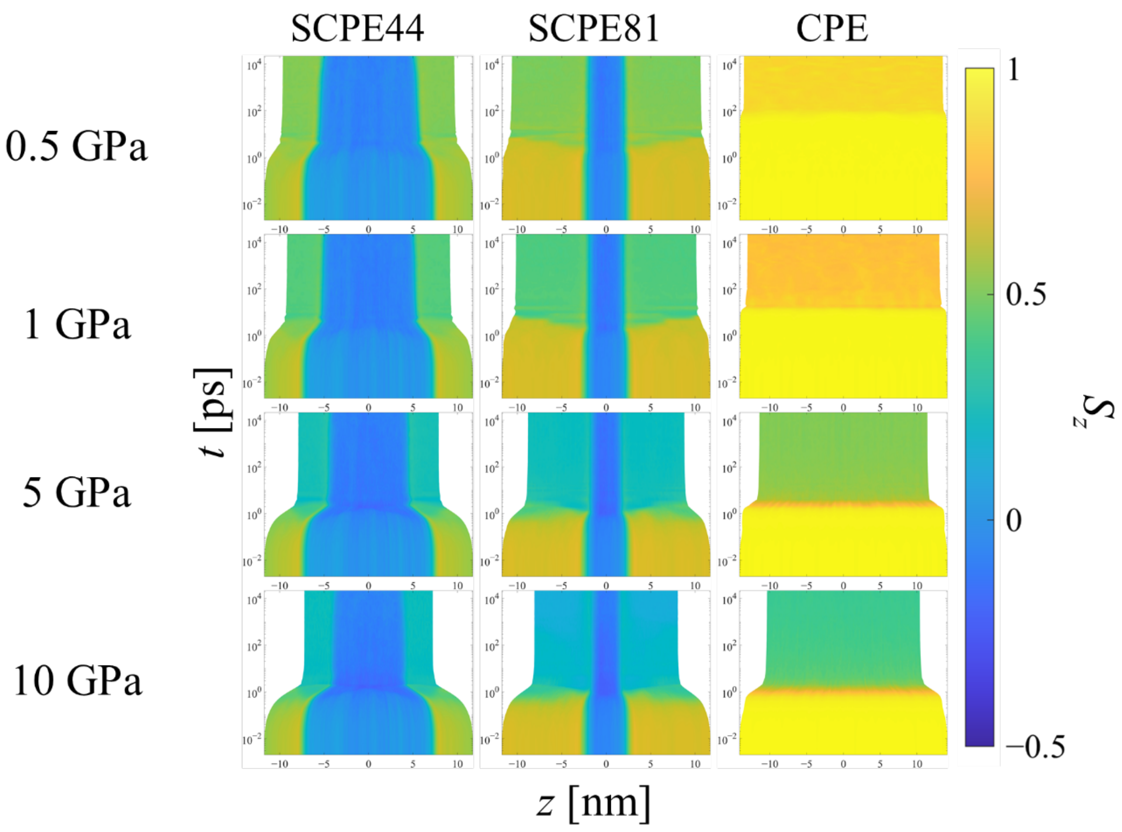

Figure S3. *Contour plots of S_z as a function of time and position along the compression axis.*

Evaluation of Clustering Methods by Silhouette Scores

In this section, mean Silhouette scores [1,2] are used to evaluate the quality of fuzzy c-means (FCM) clustering [3] applied to united atom (UA) simulation data. A per-UA Silhouette score, s, is close to 1 for UAs close to their assigned cluster, 0 for UAs that exist near the boundaries between clusters, and –1 for UAs that are very close to a cluster different from its assigned cluster. Here, distance is defined in terms of the normed difference of a per-UA datum from the centroid of a given cluster, $\|x_i - C_k\|$. Negative Silhouette scores indicate either an imperfectly converged numerical clustering attempt or a scenario where including the UA would shift the centroid of the

cluster and increase distances from the centroid, also increasing the FCM objective function. The Silhouette metric used in this work is the Fuzzy Silhouette (FS) score proposed by Campello and Hruschka [2], which extends the original formulation by Rousseeuw [1] to score fuzzy clustering data as opposed to only "hard" clustering methods like k-means. The FS metric is also used here for k-means clustering results by assigning membership binarily, i.e., $f_i^k = 0$ or 1 for all i and k. To determine which method of clustering was expected to yield the greatest Silhouette scores, the scores were averaged among the ten different configurations used for each system and at pressures of 0, 0.5, 1, 5, and 10 GPa. Input data for the Silhouette calculation were all three order parameters in addition to all four potential energy contributions, ensuring that a high-scoring clustering was able to account for more data than were input to the clustering algorithm.

The FS results are summarized in Figure S4 – Figure S8. First the FCM results are discussed, shown in Figure S4 and Figure S5. Of all the single-variable clusterings, p_2 dominated—meaning, it had greater Silhouette scores for all pressures and SCPE systems. Clustering using $\{p_2, S_z\}$ dominated clustering using p_2 alone by approximately 4–12%, and this increase was found to be statistically significant. In contrast, clustering by all three order parameters did not dominate clustering by $\{p_2, S_z\}$ despite increasing the complexity and was thus rejected as a clustering method. Clustering by either $\{p_2, v\}$ or $\{v, S_z\}$ did not dominate clustering by p_2 alone, so both methods were rejected. Clustering by $\{p_2, S_z\}$ using $n_k = 2$ dominated all methods of clustering using $n_k = 3$, implying that two clusters were always sufficient to describe the SCPE systems. Thus, clustering by $\{p_2, S_z\}$ with $n_k = 2$ dominated all methods of clustering with a fewer or equal number of variables while not being dominated by any other clustering method. Based on the previous discussion, the two most promising candidates for sets of clustering variables emerged as $\{p_2\}$ and $\{p_2, S_z\}$. However, it was determined that the marginal improvement in FS scores when including

S_z was not significant enough to warrant the additional complexity it introduced. Consequently, for the sake of clarity and efficiency, we chose to use clustering based solely on p_2 for the primary focus of this study.

Results using k-means clustering are shown in Figure S6 and Figure S7. Generally, the FS scores for FCM and k-means are comparable, but FCM data tend to have smaller uncertainties. FCM is also more appropriate for such SCPE systems because UAs near interfaces can be difficult to characterize as strictly crystalline or noncrystalline. For these reasons, we only present results from FCM clustering in the main part of this work.

Silhouette scores cannot be calculated for CPE systems when only a single cluster was used; instead, probability distributions of the three order parameters were calculated for CPE systems and observed to all be unimodal. Unimodality in and of itself is not conclusive evidence that only one population represents the data; however, it is unclear what probability distribution should be imposed on a single population of the data to test for the presence of multiple populations. Therefore, there is no direct answer in the current work of whether or not the CPE systems should be represented with multiple clusters. However, it may be verified from Figure S8 that increasing the number of clusters for n_k = 2, 3, and 4 decreases or barely changes the mean Silhouette scores in every case. Thus, the most promising options for clustering CPE seem to be n_k = 1 or 2.

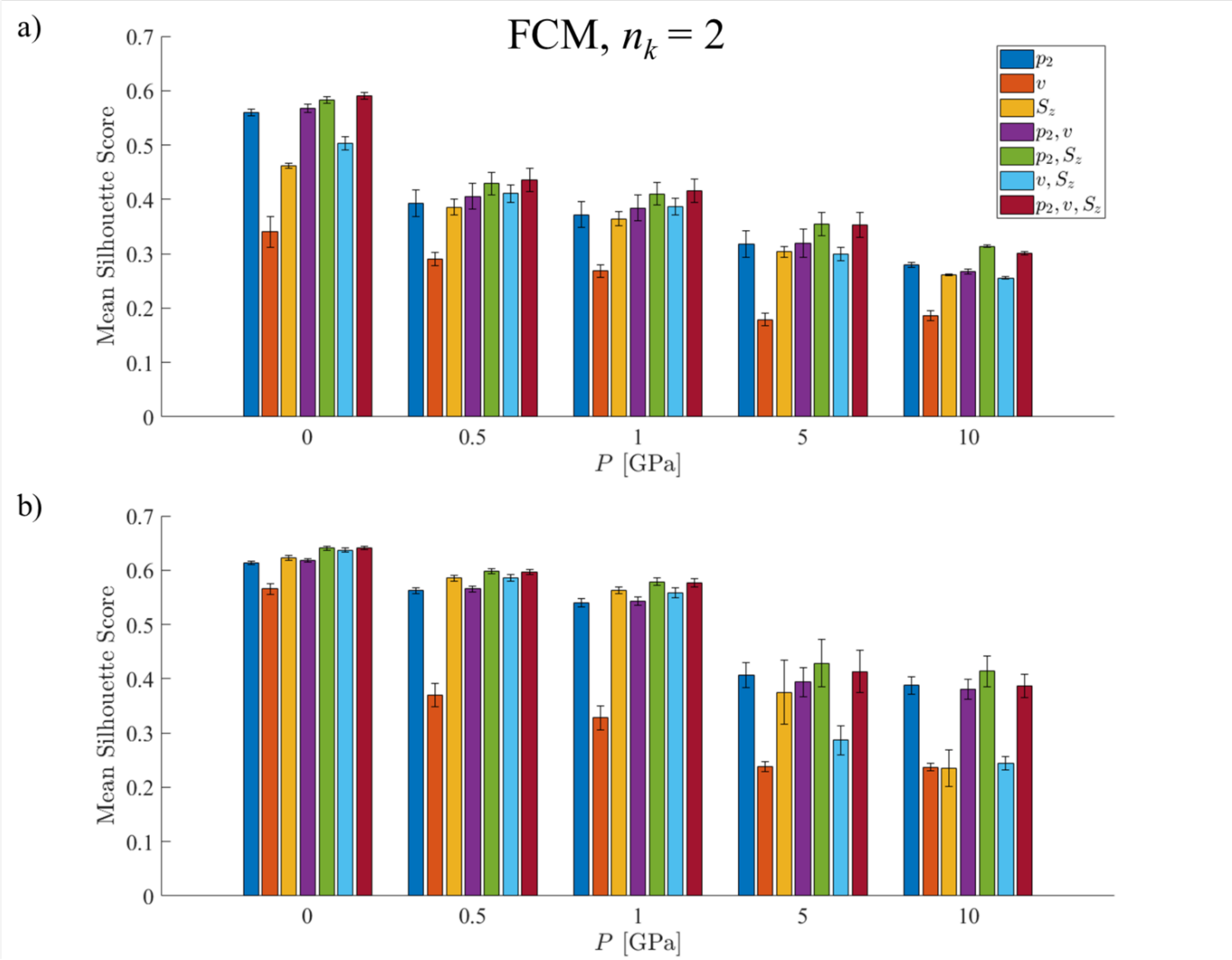

Figure S4. *Mean FS scores for FCM clustering using n_k = 2. Different bar colors represent clustering based on different subsets of the order parameters. The variables used to cluster for each color are shown in the legend. (a) shows the results for SCPE44 while (b) shows the results for SCPE81. Error bars are 3 standard errors from averaging over the ten different configurational seeds for each system.*

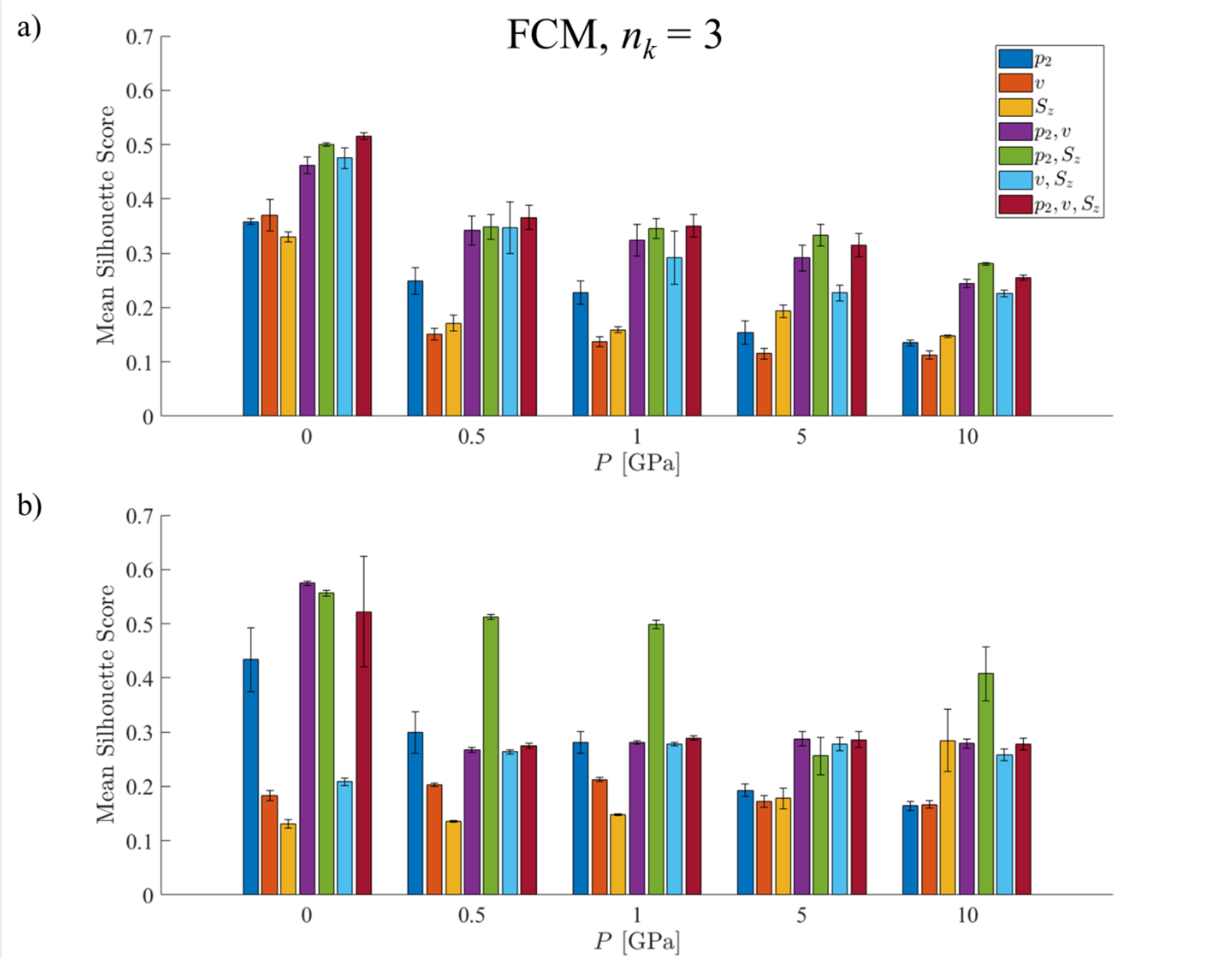

Figure S5. *Mean FS scores for FCM clustering using n_k = 3. Different bar colors represent clustering based on different subsets of the order parameters. The variables used to cluster for each color are shown in the legend. (a) shows the results for SCPE44 while (b) shows the results for SCPE81. Error bars are 3 standard errors from averaging over the ten different configurational seeds for each system.*

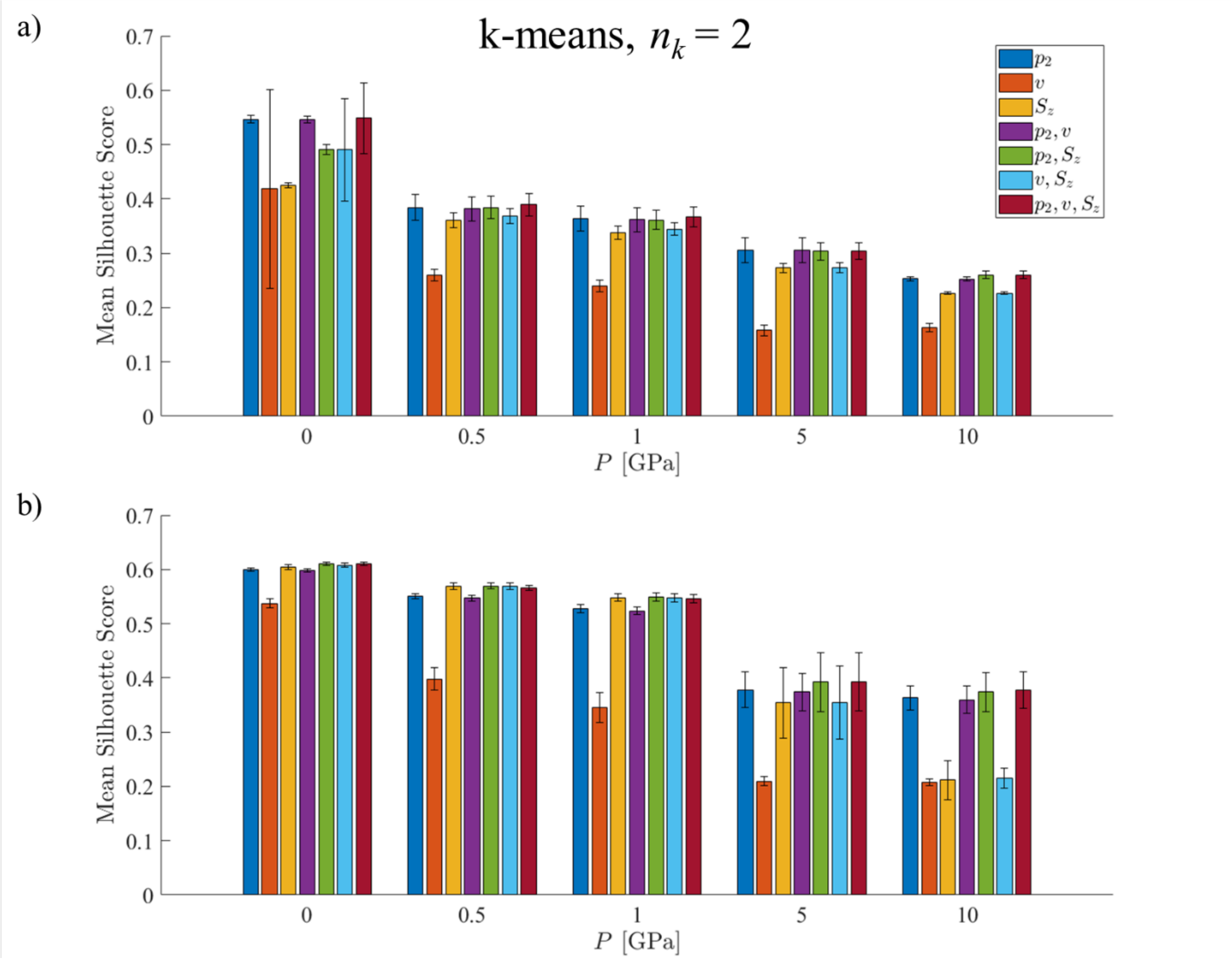

Figure S6. *Mean FS scores for k-means clustering using n_k = 2. Different bar colors represent clustering based on different subsets of the order parameters. The variables used to cluster for each color are shown in the legend. (a) shows the results for SCPE44 while (b) shows the results for SCPE81. Error bars are 3 standard errors from averaging over the ten different configurational seeds for each system.*

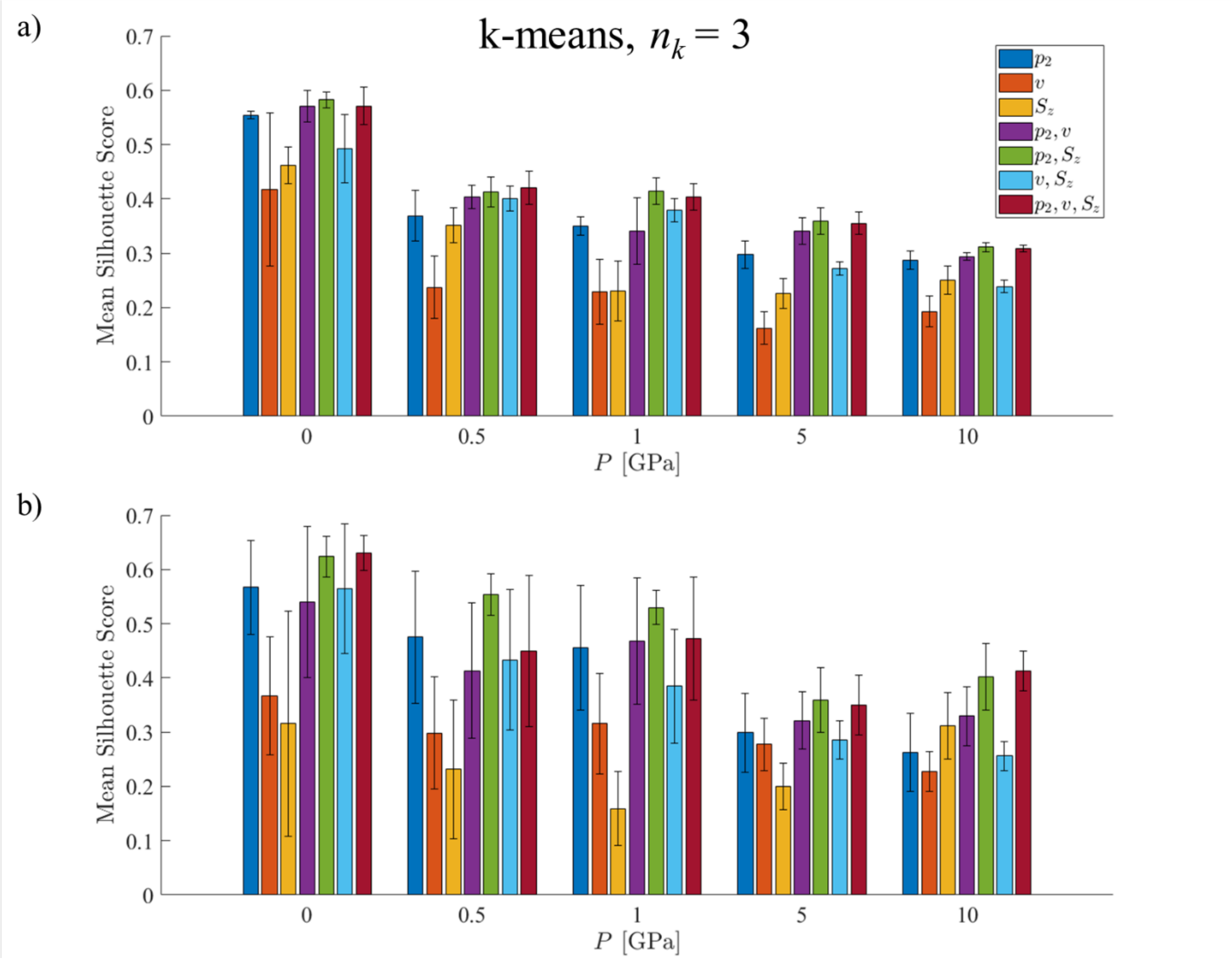

Figure S7. *Mean FS scores for k-means clustering using* $n_k = 3$*. Different bar colors represent clustering based on different subsets of the order parameters. The variables used to cluster for each color are shown in the legend. (a) shows the results for SCPE44 while (b) shows the results for SCPE81. Error bars are 3 standard errors from averaging over the ten different configurational seeds for each system.*

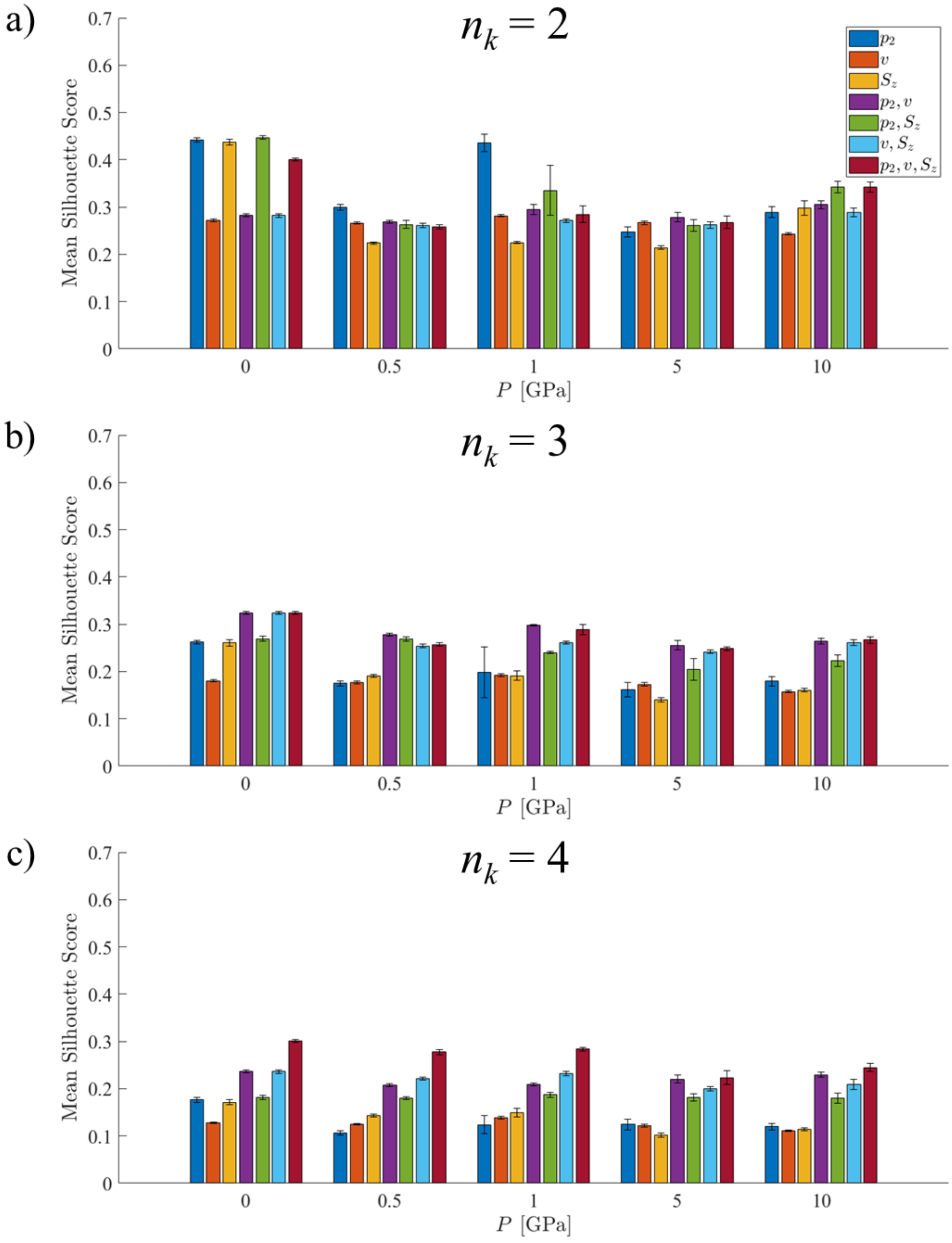

Figure S8. *For CPE systems, mean FS scores for FCM clustering using (a) n_k = 2, (b) n_k = 3, and (a) n_k = 4. Different bar colors represent clustering based on different subsets of the order parameters. The variables used to cluster for each color are shown in the legend. Error bars are 3 standard errors from averaging over the ten different starting configurations.*

References

[1] Rousseeuw, P. J. (1987). Silhouettes: a graphical aid to the interpretation and validation of cluster analysis. *Journal of computational and applied mathematics*, *20*, 53-65.

[2] Campello, R. J., & Hruschka, E. R. (2006). A fuzzy extension of the silhouette width criterion for cluster analysis. *Fuzzy Sets and Systems*, *157*(21), 2858-2875.

[3] Bezdek, J. C. (1981). *Pattern recognition with fuzzy objective function algorithms*. Springer Science & Business Media.
